# Development and validation of an interpretable machine learning model identify the lactylation-related protein SUSD3 as a prognostic and therapeutic biomarker for breast cancer

**DOI:** 10.3389/fimmu.2026.1701978

**Published:** 2026-01-27

**Authors:** Lina Tang

**Affiliations:** Institute of Trauma and Metabolism, Zhengzhou Central Hospital Affiliated to Zhengzhou University, Zhengzhou, China

**Keywords:** breast cancer, lactylation, single-cell RNA sequencing, spatial transcriptomics, machine learning, SHAP

## Abstract

**Background:**

Breast cancer is one of the most prevalent malignancies and a leading cause of cancer-related mortality among women. Lactylation, a recently recognized post-translational modification, has emerged as a significant factor in tumor biology, with increasing evidence linking it to cancer progression and immune modulation. However, the role of lactylation in tumorigenesis remains ambiguous. This raises questions about whether it serves as a primary driver or a secondary regulator during cancer development, as well as its influence on the tumor immune microenvironment and prognostic implications.

**Methods:**

This study investigates the clinical relevance of lactylation-related genes (LRGs) in breast cancer through a comprehensive analysis of extensive genomic datasets, including single-cell RNA sequencing, bulk transcriptomic data, and spatial transcriptomics from established public databases such as TISCH, TCGA, and GEO.

**Results:**

By using a combination of multiple machine-learning algorithms, we developed an effective lactylation-related signature that correlates with immune cell infiltration, chemokine expression, and tumor mutation burden. This signature proved useful in identifying breast cancer patients likely to respond to immunotherapy. Finally, we experimentally validated the quantified expression levels of hub genes in human breast samples and demonstrated the role of SUSD3.

**Conclusion:**

These findings indicate that our lactylation risk model can be used to predict the malignant progression and immune evasion of breast cancer. It is expected to become a potential therapeutic target and a diagnostic marker for breast cancer. This model also provides insights into breast cancer therapy and an effective framework for developing gene screening models applicable to other diseases and pathogenic mechanisms.

## Introduction

Breast cancer remains a leading cause of cancer-related deaths among women. The diverse molecular subtypes present considerable challenges for precision medicine, underscoring the need for tailored treatment approaches that account for tumor heterogeneity (1, 2). Despite the promise shown by recent advancements in immunotherapy, challenges such as drug resistance and treatment-related toxicities complicate the management of breast cancer, emphasizing the need for strategies that can distinguish patient responses to these therapies (1, 3–5).

Lactylation—identified by Zhao et al. in 2019—has garnered attention for its role in tumorigenesis (6–8). Studies indicate that lactylation is prevalent across various cancers, affecting processes like cell proliferation, metastasis, immune escape, drug resistance, and metabolic reprogramming of tumor cells (7). It has been reported that histone lactylation not only promotes proliferation, metastasis, and invasion but also contributes to targeted therapy resistance in clear cell renal cell carcinoma (ccRCC) (9), colorectal cancer (CRC) (10, 11), ocular melanoma (12), non-small cell lung cancer (13), breast cancer (14), and liver cancer (15, 16). Non-histone lactylation has also been validated to promote tumor progression and affect treatment resistance in hepatocellular carcinoma (17, 18), pancreatic ductal adenocarcinoma (19), prostate cancer (20), CRC (21–23), glioblastoma (24), breast cancer (25), and gastric cancer (10, 26, 27). Moreover, lactylation is involved in the regulation of tumor metabolism by promoting the expression of enzymes related to the TCA cycle, enhancing the glucose uptake ability of tumor cells, and further exacerbating metabolic disorders within tumors (7). While the significance of lactylation in cancer is increasingly recognized, its precise role in breast cancer, whether as a major driver or a minor regulator, remains poorly understood. At present, predictive models for assessing the prognostic significance of LRGs in breast cancer are still lacking. Thus, developing effective models of lactylation could provide a promising new approach for identifying potential biomarkers for the diagnosis and treatment of breast cancer.

In this study, we integrated scRNA-seq with multi-omics approaches to elucidate the cellular landscape of breast cancer and its association with lactylation-related genes. By identifying differentially expressed genes (DEGs) across lactylation clusters, we constructed a prognostic model that demonstrates improved accuracy compared to existing frameworks by utilizing machine learning algorithms. Our analysis reveals significant correlations between lactylation signatures and immune infiltration, clinical characteristics, and patient survival outcomes through cluster analysis, enrichment analysis, and survival analysis. Additionally, we assessed the potential of these predictive models to identify breast cancer patients who are likely to benefit from immunotherapy by examining the interactions among immune components within the context of breast cancer. Furthermore, we investigated the spatial relationship between SUSD3 and fibroblasts in breast cancer patients using spatial transcriptomics. Collectively, our work provides innovative and comprehensive insights into the role of lactylation in breast cancer and its implications for personalized treatment strategies, laying the groundwork for a deeper understanding of the influence of lactylation on clinical outcomes and the tumor microenvironment.

## Materials and methods

### Data collection and processing

Single-cell RNA sequencing (scRNA-seq) data specific to breast cancer were sourced from the TISCH database (http://tisch.comp-genomics.org/) with the following datasets: BRCA_GSE161529, BRCA_EMTAB8107, and BRCA_GSE148673. The R package “CellChat” was utilized to analyze intercellular communication by examining ligand-receptor interactions, which allowed for the prediction of potential communication networks within the tumor microenvironment.

Bulk RNA-seq data along with clinical information were obtained from the GEO database (https://www.ncbi.nlm.nih.gov/geo/), specifically from the following datasets: GSE162228 (133 samples and 23361 genes), GSE20685 (327 samples and 23342 genes), GSE42568 (104 samples and 21989 genes), GSE58812 (107 samples and 23374 genes), and GSE88770 (117 samples and 23324 genes). Additionally, data from The Cancer Genome Atlas (TCGA) (https://www.cancer.gov/ccg/research/genome-sequencing/tcga) were included, comprising 1072 samples and 41521 genes. To facilitate comprehensive analysis, the datasets were merged, and inter-batch differences were adjusted using the R packages limma and sva. This preprocessing resulted in a final dataset of 19,144 genes across 1,845 samples.

LRGs were identified from literature (PMID:37242427, PMID:35761067, and PMID:36092712). A total of 336 lactylation-related genes were identified, of which 206 were found to overlap with the gene expression profiles present in the RNA-seq datasets utilized.

### Scoring of lactylation across different cell types

To evaluate the lactylation score among different cell types, the R package GSVA (Gene Set Variation Analysis) was employed. This analysis categorized 13 distinct cell types into high- and low-score groups based on their lactylation scores.

Following this, hallmark pathways were retrieved from the MsigDB database. The GSVA package was again utilized to assess both lactylation and hallmark pathway scores, facilitating an exploration of the correlation between lactylation and hallmark pathways. The results of this analysis are visually represented in a heatmap.

Further, the CellChat package was employed to analyze receptor-ligand signaling interactions between the high- and low-lactylation score groups, providing insights into the communication dynamics within the tumor microenvironment.

### Construct a patient-specific classification of breast cancer based on LPAGs

To identify significant lactylation prognosis-associated genes (LPAGs), the R packages “survival” and “survminer,” alongside univariate COX regression analysis. A total of 35 genes were identified, and the R package “ConsensusClusterPlus” was utilized to classify breast cancer patients into 3 clusters. A heatmap generated using the R package “pheatmap” visually illustrates the correlation between clinical characteristics and gene expression across the 3 clusters. To assess functional differences among the 3 clusters, the R package “GSVA” was employed alongside hallmark pathways from the MsigDB database. The results of this functional analysis were also visualized un a heatmap created using the R package “pheatmap”.

### Mutation analysis of different lactylation clusters

To investigate the mutation landscape across different lactylation clusters, the R package “maftools” was utilized to generate a waterfall plot that visually represent the mutation frequencies and types in patients corresponding to each lactylation cluster.

### Immune infiltration analysis

To assess immune infiltration levels across the different lactylation clusters, single-sample gene set enrichment analysis (ssGSEA) was performed. Additionally, the R package “IOBR” was utilized in conjunction with 7 established algorithms: MCPcounter, EPIC, CIBERSORT, IPS, quanTIseq, ESTIMATE, and TIMER. These tolls were employed to evaluate the proportions of various immune cell types among the lactylation clusters.

### Machine learning-based integration constructs a prognostic model of DEGs

Differentially expressed genes (DEGs) were identified across 3 different lactylation clusters with a |LogFC|>0.5 and an adjusted P <0.05, resulting in a total of 5,640 DEGs after intersection. Subsequent functional analyses, including Gene Ontology (GO) and Kyoto Encyclopedia of Genes and Genomes (KEGG), were conducted using the R package “clusterprofiler”.

Univariate Cox regression analysis was performed on the identified 5640 DEGs using data from several cohorts, including TCGA and multiple GEO dataset (GSE20685, GSE42568, GSE58812, GSE88770, and GSE162228). The analysis, conducted with the R packages “survival” and “survminer”, identified 14 genes with significant prognostic value (p < 0.05) that were selected for further investigation.

To develop a robust predictive model, a combination of 10 classical machine learning algorithms was integrated, including: random survival forest (RSF), least absolute shrinkage and selection operator (LASSO), gradient boosting machine (GBM), survival support vector machine (Survival-SVM), supervised principal components (SuperPC), ridge regression, partial least squares regression for Cox (plsRcox), CoxBoost, Stepwise Cox, and elastic network (Enet). Among these, RSF, LASSO, CoxBoost, and Stepwise Cox effectively performed dimensionality reduction and variable screening, resulting in 99 combinations of machine learning algorithms. The TCGA dataset served as the training cohort, while the remaining five cohorts were utilized for validation purposes. Harrell’s concordance index (C-index) was calculated across all validation datasets, identifying RSF as the most effective model due to its highest average C-index.

The training and validation cohorts were categorized into low- and high-risk groups based on the median risk score derived from the model. To assess the accuracy of the lactylation-associated genes prognostic index (LAGPI) and the predictive ability of the signature biomarkers, the receiver operating characteristic (ROC) curve and area under the curve (AUC) calculation were utilized using the R package “pROC”. Additionally, time-dependent ROC curves were plotted using the R package “timeROC” to evaluate the predictive efficacy of LAGPI for survival outcomes.

The SHapley Additive exPlanations (SHAP) method was employed to visually analyze the contribution and influence of each feature in the model’s predictions.

Finally, dependency scores for genes affecting cell viability were generated using the CERES algorithm available on the DepMap website (https://depmap.org/portal/).

### Drug sensitivity analysis

The R package “oncoPredict” was utilized to calculate the half maximal inhibitory concentration (IC50) of various drugs in both high- and low-risk groups.

### Spatial transcriptomics analysis

Spatial transcriptomics data for SUSD3 in breast cancer were obtained from the CROST database (https://ngdc.cncb.ac.cn/crost/home) and analyzed using the R packages “Seurat” and “semla”.

### Statistical analysis

All experimental results were performed with a minimum of 3 replicates. Data are presented as mean ± SD and analyzed parametrically using GraphPad Prism (San Diego, CA). Statistical significance between two groups was assessed using the unpaired Student’s t‐test. Ordinary one‐way ANOVA was employed to analyze among more than two groups. Two‐way ANOVA was utilized to assess cell proliferation at different timepoints. Differences between means with P < 0.05 were considered significant.

## Results

### Large-scale integration of a single-cell RNA sequencing atlas reveals cell diversity in breast tumors

**Figure 1:**
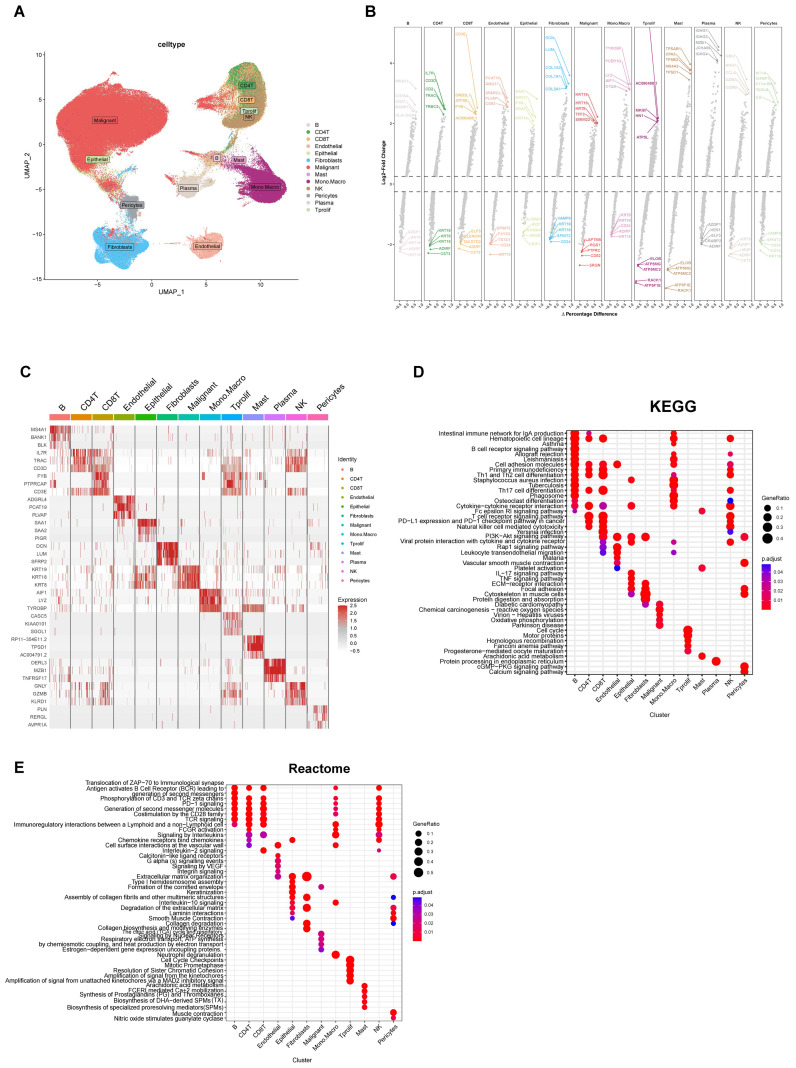
Cell diversity and functional enrichment. **(A)** UMAP visualization was employed to illustrate 13 distinct cell types. **(B)** The FindAllMarkers function was utilized to identify differentially expressed genes across various cell types, and the top five genes with both high and low expression levels in each cell type were displayed. **(C)** Marker genes for each cell type were identified using the R package “COSG”, and a heatmap was generated to depict the expression levels of the top 3 marker genes. **(D, E)** Based on the results obtained from COSG, the top100 genes for each cell type were selected for enrichment analysis using the R package “clusterprofiler”, with the results of the KEGG **(D)** and Reactome I pathways presented.

To elucidate the cellular composition and heterogeneity in breast cancer, we collected and analyzed scRNA-seq data from 3 datasets of breast cancer (BRCA_GSE161529, BRCA_EMTAB8107, BRCA_GSE148673) available in the TISCH database. Cells were categorized into 13 distinct cell types based on specific gene expression patterns, which includ B cells, CD4 T cells, CD8 T cells, endothelial cells, epithelial cells, malignant cells, mast cells, monocytes/macrophages, NK cells, pericytes, plasma B cells, and prolif-T (proliferating T) cells (**Figure 1A**). Differential expression analysis was performed to identify differentially expressed genes for each cluster across all 13 cell types, as illustrated in **Figure 1B**. The heatmap plot depicted the top 3 marker genes for each cell population (**Figure 1C**). Functional enrichment analysis revealed significant differences among the cell types (**Figures 1D, E**).

**Figure 2:**
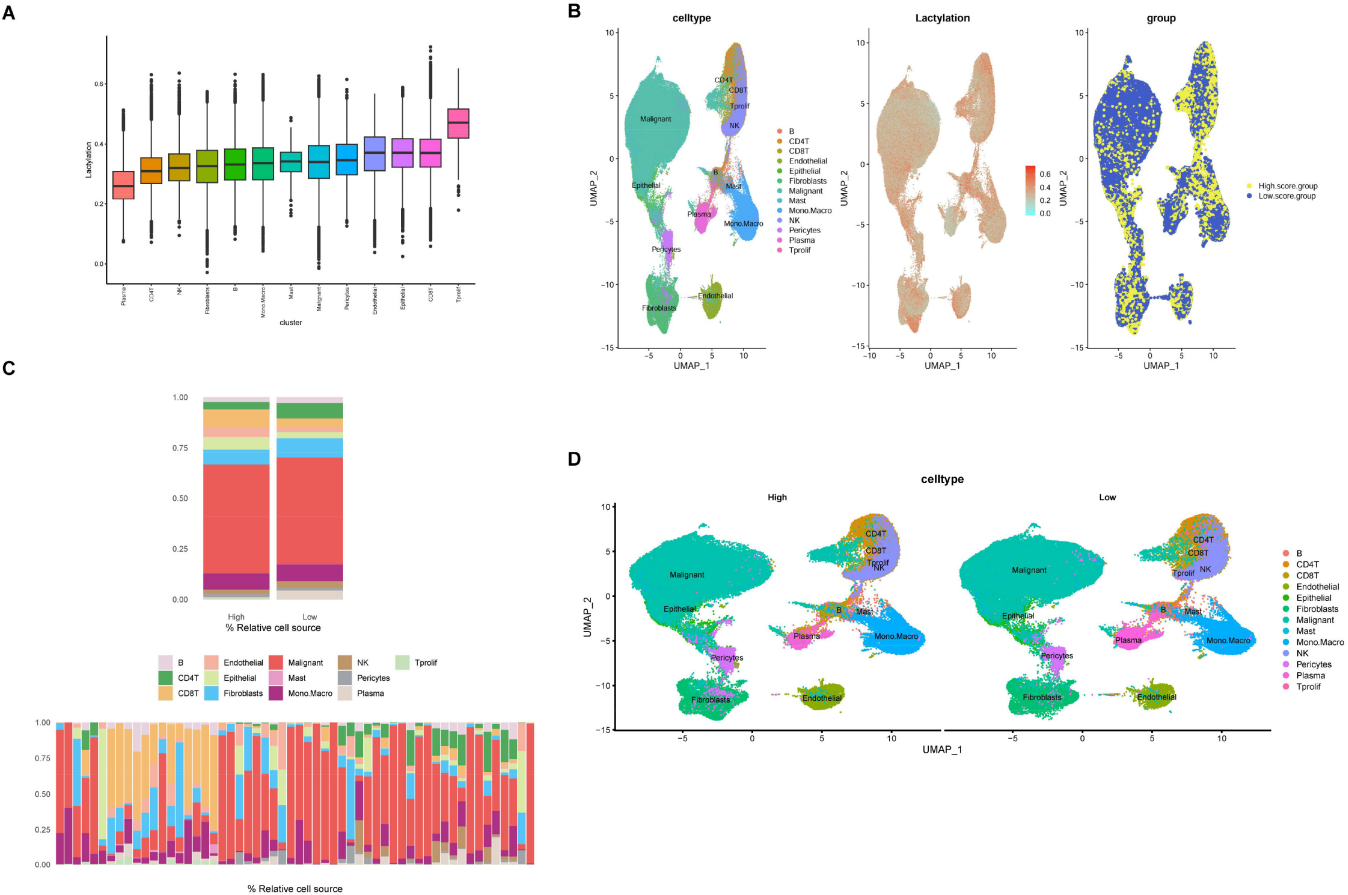
Lactylation profiling of breast cancer patients based on single cell-sequencing. **(A)** Each cell was scored based on the lactylation gene set using the R package “GSVA,” assessing the expression levels of the gene set across different cell types. **(B)** UMAP showed the lactylation gene set scores across each cell type, and cells were divided into high-score and low-score groups. **(C)** The proportion of cells for each cell type was compared between the high- and low-score groups based on lactylation gene set scoring. **(D)** A UMAP plot was generated to visualize the distribution of cell types between the high- and low-score groups.

A total of 336 lactylation-related genes (LRGs) were identified from published literature (PMID:37242427, 35761067, 36092712). Following the intersection of these with the scRNA-seq gene expression profile, 206 genes were selected to evaluate lactylation scores across the various cell types (**Figure 2A**). Cells were categorized into high- and low-score groups based on the median lactylation scores (**Figure 2B**). As illustrated in **Figure 2B**, plasma B cells exhibited the lowest activity, as shown by the UMAP plot. Furthermore, significant differences in composition and distribution among cell types were observed between the high- and low-score groups (**Figures 2C, D**). Specifically, CD8 T cells, endothelial cells, epithelial cells, and pericytes displayed higher levels of lactylation, whereas CD 4T cells, fibroblasts, NK cells, and plasma cells exhibited comparatively lower lactylation levels.

To further elucidate the key pathways related to lactylation, each cell was scored using the R-package GSVA based on the MSigDB dataset, and the correlation between HALLMARK pathway scores and lactylation levels across various cell types was analyzed. We found that the “MYC TARGETS V”, “MTORC1 SIGNALING”, “E2F TARGETS”, and “G2M CHECKPOINT” pathways exhibited the strongest correlation across all cell types (**Supplementary Figure S1**).

Further analysis of cell communication revealed that the high-score group exhibited significantly greater intercellular interactions compared to the low-score group, with endothelial cells and monocytes/macrophages serving as central communicators (**Supplementary Figures S2A, B**). The strength of both incoming and outgoing signals was notably higher in the high-score cohort, as illustrated in **Supplementary Figures S2C** and **S2D**. The heatmaps showed the strength of incoming and outgoing signal for each signaling pathway among different cell types (**Supplementary Figure S2E**).

### Screening and characterization of genes associated with lactylation in breast cancer

**Figure 3:**
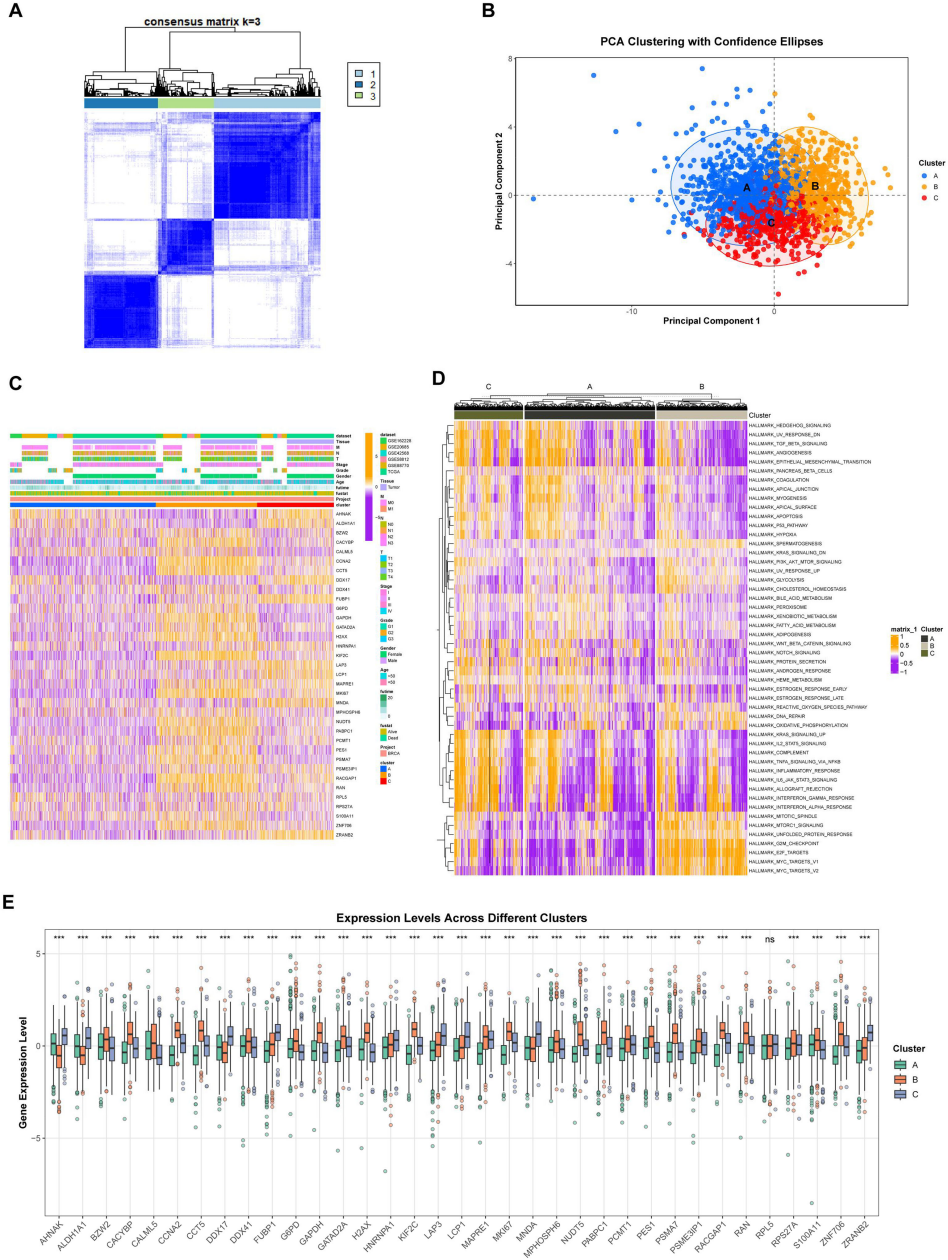
Consensus clustering analysis of patient-specific classification of breast cancer based on 35 LPAGs. **(A)** A heatmap described the unsupervised consensus clustering solution when k = 3. **(B)** A PCA plot showed the distribution of samples across the 3 clusters. **(C)** A heatmap showed the characterization of clinicopathologic features and the expression of LPAGs within the 3 lactylation clusters. **(D)** The GSVA comparison of biological pathways among the 3 lactylation clusters in breast cancer is presented. **(E)** Differences in the expression of 35 LPAGs across the various clusters are shown. Ns p>0.05, ***p<0.001.

To identify genes associated with differential lactylation in breast cancer, 6 breast cancer-related datasets (GSE162228, GSE20685, GSE42568, GSE58812, GSE88770, and TCGA) were obtained. Differential analysis was conducted using the “limma” package, encompassing 1860 samples and 19144 genes. The intersection of these genes with 336 lactylation-related genes yielded a total of 206 genes. Further univariate Cox analysis identified 35 lactylation prognosis-associated genes (LPAGs) (Cox p<0.01), as shown in **Supplementary Figure S3**. Unsupervised consensus clustering was employed to develop a patient-specific classification of breast cancer based on the 35 LPAGs. Using the optimal classification with k = 3, breast cancer samples were clustered into 3 groups: cluster A (n = 837, 45%), cluster B (n = 582, 31.3%), and cluster C (n = 441, 23.7%) (**Figure 3A**). Principal component analysis clearly distinguished breast cancer samples across LPAG clusters A, B, and C (**Figure 3B**). Additionally, clinicopathological features and the expression levels of the 35 LPAGs were visualized in a heatmap, with most differentially expressed genes occurring in gene cluster B (**Figure 3C**). To uncover the biological pathways related to these 3 clusters, each cell was scored using the R package GSVA based on the MSigDB dataset, revealing that cluster C was markedly enriched in “MITOTIC SPINDLE”, “MTORC1 SIGNALING”, “UNFOLODED PROTEIN RESPONSE”, “G2M CHECKPOINT”, “E2F TARGETS”, “MYC TARGETS V1”, and “MYC TARGETS V2” (**Figure 3D**). Furthermore, the differential expression levels of the 35 LAPGs across the 3 clusters are presented in **Figure 3E**, indicating that, apart from AHNAK, ALDH1A1, DDX17, FUBP1, HNRNPA1, LAP3, LCP1, MNDA, RPL5, and ZRANB2, which were highly expressed in cluster C, the remaining genes exhibited higher expression in cluster B.

Given the significant role of gene mutations in tumor progression, the maftools algorithm was utilized to investigate the top ten genes with the highest mutation rates across the 3 clusters. Notably, the mutation rates of TP53 (cluster B, 49%; cluster A, 19%; cluster C, 14%) and TIN (cluster B, 19%; cluster A, 15%; cluster C, 13%) exhibited higher mutation rates in cluster B than in clusters A and C (**Supplementary Figures S4A–C**). The differences in gene mutations between each pair of clusters are illustrated in **Supplementary Figures S4D**, **S4E**, and **S4F**.

### Landscape of immune cell infiltration and correlation

The differences in immune cell infiltration among the 3 clusters were assessed using the ssGSEA algorithm, which provided valuable insights into the composition and functionality of immune cells. Initially, the immune infiltration scores of 28 immune cell types across the 3 clusters were compared. Cluster C exhibited the highest infiltration scores for 23 immune cell types, including activated B cell, activated CD8 T cell, activated dendritic cell, CD56bright natural killer cell, CD56 dim killer cell, central memory CD4 T cell, central memory CD8 T cell, effector memory CD4 T cell, effector memory CD 8 T cell, eosinophil, immature B cell, immature dendritic cell, macrophage, mast cell, memory B cell, myeloid derived suppressor cell, natural killer cell, nature killer T cell, plasmacytoid dendritic cell, regulatory T cell, T type 1 T helper cell, follicular helper cell, and type 2 T helper cell. In contrast, the highest infiltration scores for neutrophils and Th17 cells were observed in cluster A, while the highest scores for activated CD4 T cell and gamma delta T cell were noted in cluster B (**Supplementary Figure S5A**). To minimize potential bias from the analytical algorithm, further evaluations of immune infiltration levels among the 3 subtypes were conducted using 7 different immune infiltration assessment methods, with the results presented in a heatmap (**Supplementary Figure S5B**).

### Machine learning-based integration constructs a prognostic model of DEGs

Distinct sets of DEGs were identified among the 3 subtypes, resulting in a total of 5640 DEGs (with a threshold of |logFC|>0.5 and an adjusted p<0.05). To explore the associated biological pathways, Gene Ontology (GO) functional enrichment and KEGG pathway enrichment analyses were performed based on the DEGs. The GO enrichment analysis indicated that the DEGs were related to RNA splicing, DNA-binding transcription factor binding, spindle, and chromosomal region (**Supplementary Figure S6A**). The KEGG analysis revealed that the DEGs were enriched in the PI3K-AKT signaling pathway, MAPK signaling pathway, cell cycle, and cellular senescence (**Supplementary Figure S6B**).

To assess the prognostic value of the DEGs across the 3 clusters in breast cancer, a risk model was constructed. Univariate regression analysis of all 5640 subtype-related genes revealed that 14 of these genes were significantly associated with prognosis across at least 5 breast cancer datasets (**Supplementary Figure S7**).

**Figure 4:**
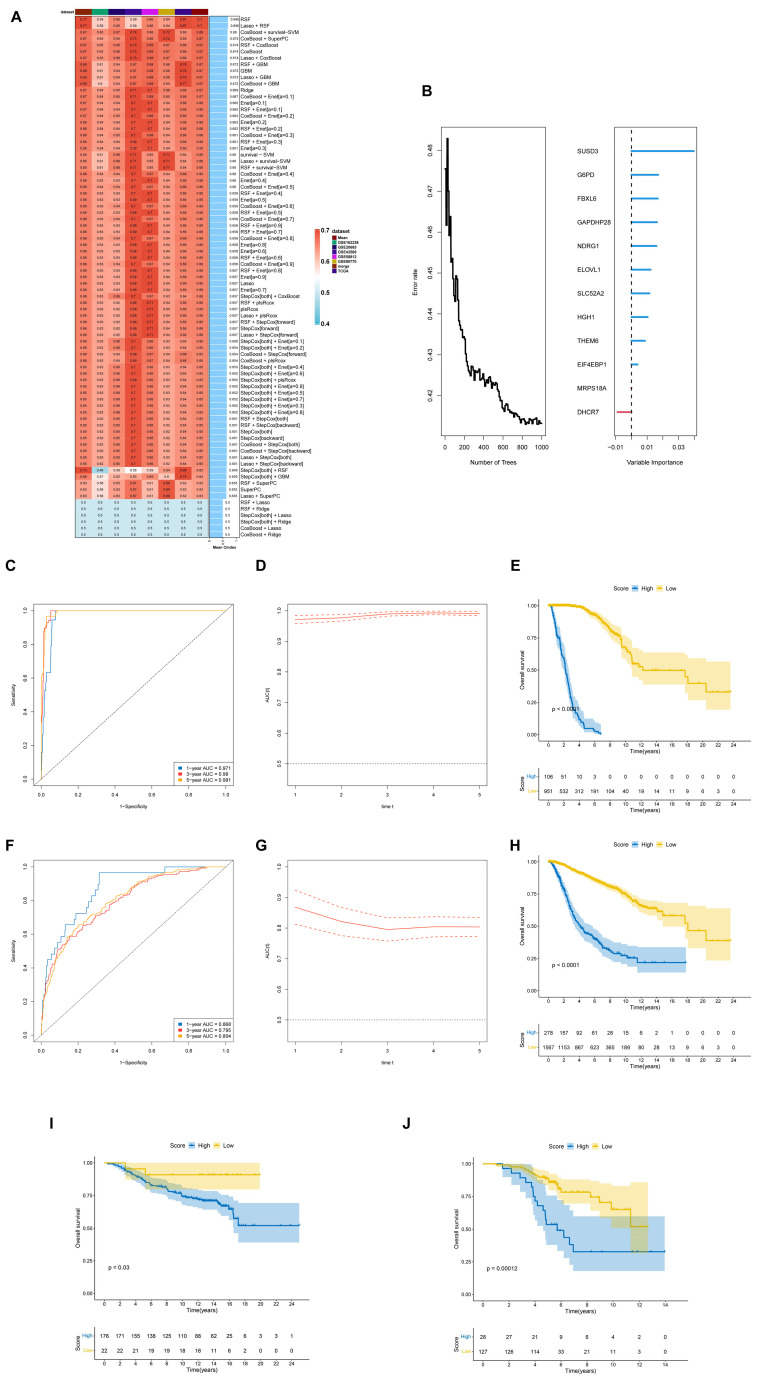
Machine learning-based integration constructs a prognostic model of DEGs. **(A)** A total of 10 classical algorithms were combined with other algorithms to create 99 machine-learning algorithm combinations for constructing a lactylation-associated genes prognostic index (LAGPI). The C-index for each model was calculated across all validation datasets, with the model developed using the random forest (RSF) method identified as the most effective. **(B)** The results of the selected random forest model were displayed, and scoring models were constructed based on the importance of the variables identified. **(C–E)** The prognostic score was predicted using RSF within the TCGA training set. The ROC curve was generated, where the X-axis represents “1-specificity”, and the Y-axis represents “sensitivity”. Prediction for different genes yield varying “sensitivity and specificity” and “1-specificity”. Consequently, different genes can be predicted with diverse sensitivities, specificities, and areas under the ROC curves (AUC), reflecting the predictive power of the genes concerning diseases occurrence. **I** The time-dependent ROC curve and **(F)** the survival curve was also included. **F-H.** The prognostic score was predicted by using RSF within the merged validation cohort. **(F)** The ROC curve, **(G)** the time-dependent ROC curve, and **(G)** the survival curve was illustrated. **(I)** The survival curve was conducted using GSE7390. **(J)** The survival curve was conducted using GSE9893.

**Figure 5:**
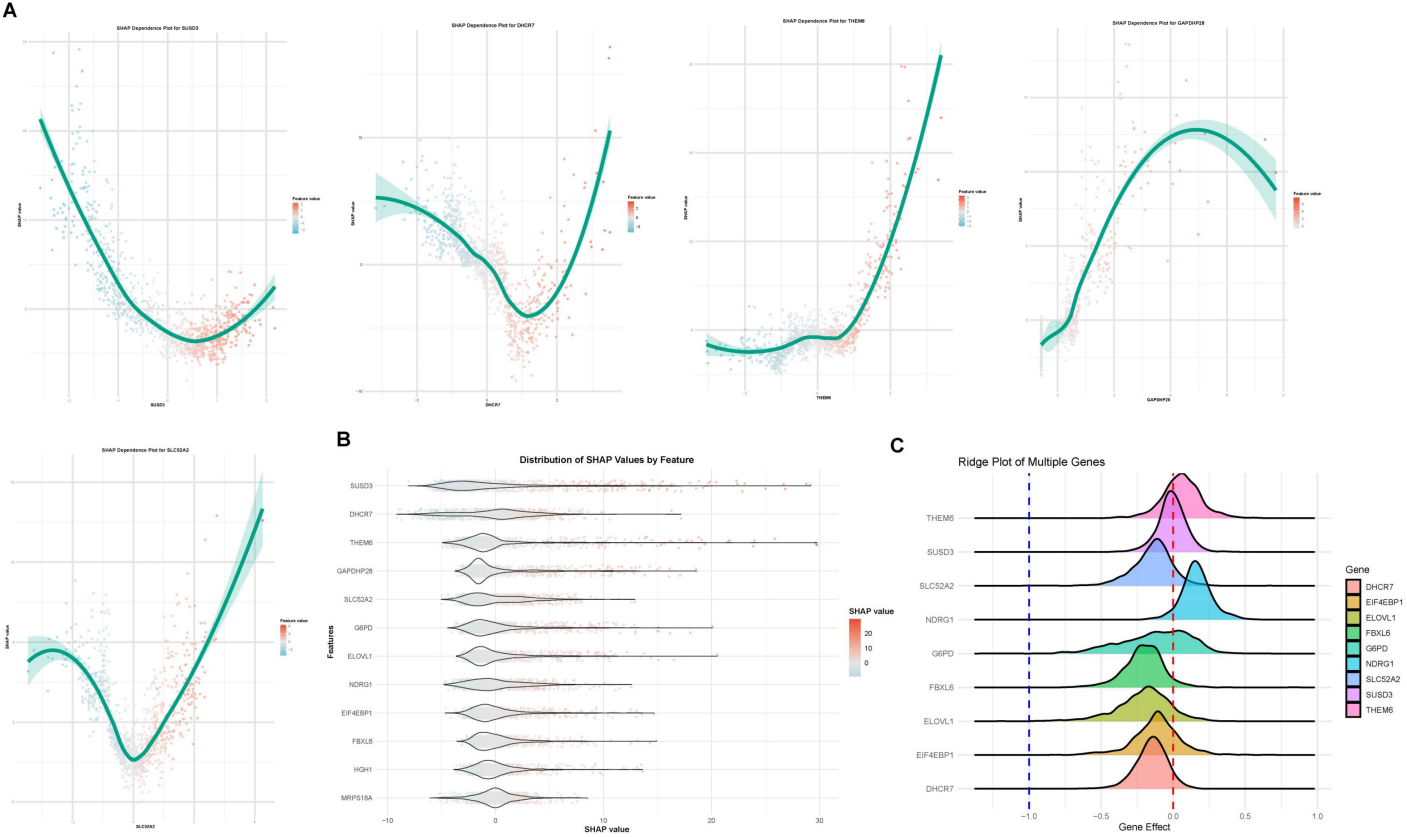
SHAP analysis identifies key DEGs affecting lactylation risk prediction models of breast cancer. **(A)** SHAP (SHapley Additive exPlanations) is a tool utilized to elucidate predictions made by machine learning models, aiming to assign an “importance” score to each feature, which reflects the feature’s contribution to the model’s predictions. Five of these genes were presented. **(B)** The distribution of SHAP values by feature was illustrated. **(C)** DepMap (The Cancer Dependency Map) analysis was employed to investigate the influence of genes on cell survival and function. The distribution of the effects of key genes was presented. Genes positioned further to the left exhibit a stronger inhibitory effect on cell proliferation following knockout.

These 14 genes were subsequently subjected to various machine learning algorithms to construct a lactylation-associated genes prognostic index (LAGPI). The TCGA dataset was utilized as the training cohort, while 5 other cohorts served as validation cohorts. The optimal model, identified through the average concordance index (C-index) across the 5 cohorts, was the random survival forest (RSF) model, achieving the highest average C-index of approximately 0.696 (**Figure 4A**). Following this, prognostic models were developed for the identified prognosis-related genes, demonstrating robust predictive capability in the RSF model (**Figure 4B**). The final prognostic model comprised 13 genes, as illustrated in **Figure 5B**, including SUSD3, G6PD, FBXL6, GAPDHP28, NDRG1, ELOVL1, SLC52A2, HGH1, THEM6, ELF4EBP1, MRPS18A, and DHCR7.

To emphasize the prognostic accuracy of the model, receiver operating characteristic (ROC) analysis and time-dependent ROC analysis were performed using both the training and the merged validation cohorts. The Area Under Curve (AUC) for the TCGA group was estimated to be 0.971, 0.99, and 0.991 for 1, 3, and 5 years, respectively (**Figures 4C, D**). In contrast, for the merged validation cohorts, the AUC was estimated at 0.868, 0.795, and 0.804 for 1, 3, and 5 years, respectively (**Figures 4F, G**). Furthermore, curve analysis was conducted for both the training and merged validation cohorts, stratifying patients into high- and low-risk groups based on median scores. The survival curve for the training group indicated that the high-risk group experienced poorer survival outcomes, a finding corroborated by the merged validation cohorts (**Figures 4E, H**).

Additionally, we evaluated the prognostic performance of LAGPI in a completely new breast cancer dataset that was not involved in model training, demonstrating its general applicability. In this analysis, the high-risk group again exhibited poorer survival outcomes, further confirming the robustness of the LAGPI in predicting the prognosis of breast cancer patients (**Figures 4I, J**).

### SHAP analysis identifies key DEGs affecting breast cancer prediction models

The SHAP (Shapley Additive exPlanations) analysis was conducted to evaluate the significance and effects of 13 DEGs identified through the RSF model-specifically, DHCR7, SUSD3, G6PD, FBXL6, GAPDHP28, NDRG1, ELOVL1, SLC52A2, HGH1, THEM6, ELF4EBP1, and MRPS18A-on the predictive performance of machine learning models. Visualizations of SHAP values provided further insights into the contributions of these genes. The results revealed that the top 5 influential genes were SUSD3, which emerged as the most significant, followed by DHCR7, THEM6, GAPDHP28, and SLC52A2 (**Figure 5A**). Additionally, **Figure 5B** illustrated the distribution of SHAP values across features, highlighting a strong correlation between higher expression levels of SUSD3 and positive predictions, thereby underscoring its vital role in the model’s decision-making process. Using data from the DepMap (The Cancer Dependency Map) database, we investigated gene dependency across various cell lines through the Chronos score generated by CRISPR-Cas9 technology. The findings indicated that FBXL6, ELOVL1, ELF4EBP1, and DHCR7 were essential genes for cellular function and may promote cell proliferation. In contrast, SUSD3 and G6PD showed no significant effect on cell proliferation, while THEM6 and NDRG1 were associated with the inhibition of cell proliferation (**Figure 5C**). In summary, the SHAP analysis confirms the substantial impact of the DEGs identified by the RSF, particularly SUSD3, on the predictive accuracy of the model, highlighting their potential as biomarkers for diagnostic and therapeutic applications.

### The relationships between clinicopathological features and risk scores

Given that various clinicopathological features exert differing effects on disease prognosis, we explored the distribution of these features within the RSF model to elucidate the relationship between risk scores and clinical characteristics. The results indicated a significant correlation between risk scores and tumor progression. Notable differences in risk scores were observed across various grade levels (**Supplementary Figure S8A**), M category (**Supplementary Figure S8B**), N category (**Supplementary Figure S8C**), overall survival (OS) (**Supplementary Figure S8D**), stage level (**Supplementary Figure S8E**), and T category (**Supplementary Figure S8F**). These findings indicate that the high-risk group is characterized by a more advanced TNM stage, higher tumor grade, and poorer survival outcomes.

Subsequently, we performed univariate and multivariate Cox regression analyses to assess the predictive efficacy of the risk score in conjunction with other clinical features in breast cancer patients. The univariate Cox regression analysis revealed that risk score, grade levels, T category, N category, and M category were significantly associated with the prognosis of breast cancer patients (**Supplementary Figure S9A**). Furthermore, the multivariate Cox regression analysis indicated that age and risk score were independent prognostic factors for breast cancer patients (**Supplementary Figure S9B**).

### Functional enrichment analysis of the risk model

To further investigate the underlying mechanisms contributing to the disparate outcomes observed between high-risk and low-risk groups based on the risk score, we identified DEGs between these groups and analyzed the correlation between risk scores and DEGs. The heatmap in **Supplementary Figure S10A** illustrates the top 50 genes that are positively correlated with risk scores, while **Supplementary Figure S10B** displays the top 50 genes that are negatively correlated. Subsequently, GSEA analysis was conducted to explore GO, KEGG, and Reactome terms associated with the positively correlated top 50 genes. The significantly enriched GO terms included fat cell differentiation, regulation of phosphatidylinositol 3-kinase signaling, blood vessel development, and vasculature development. The significantly enriched KEGG terms comprised cytokine-cytokine receptor interaction, regulation of lipolysis in adipocytes, Ras signaling pathway, and Th1 and Th2 cell differentiation. Additionally, the significantly enriched Reactome terms included synthesis of DNA, G1/S transition, formation of the cornified envelope, and mitochondrial translation elongation (**Supplementary Figure S10C**).

### The correlation between risk score and stemness

Stemness refers to the capacity of normal cells to differentiate from their origin into various cell types, contributing to the development of the human organism. The gradual loss of differentiation potential and the acquisition of stem cell-like characteristics are primary factors driving tumor progression (28, 29). This study further investigates the relationship between risk score and stemness features, which influence tumor immunogenicity and susceptibility to immunotherapy. Two independent indices, mDNAsi and mRNAsi, were used to quantify the degree of differentiation and stemness in cancer cells. The findings indicated that both mDNAsi (**Supplementary Figure S11A**) and mRNAsi (**Supplementary Figure S11B**) were significantly positively correlated with the risk score.

Additionally, an analysis of 26 stem gene sets was conducted to assess the distribution of stemness scores across high-risk and low-risk groups, revealing that the high-risk group exhibited higher stemness scores (**Supplementary Figure S11C**).

### Relationships between risk score and mutations

To investigate the differences in genomic mutation frequencies between the high- and low-risk groups, mutation landscapes were illustrated for both groups. The results highlighted the most frequently mutated genes within each group. TP53 was identified as the most commonly mutated gene in the high-risk group, while PIK3CA was the most prevalent mutation in the low-risk group. Additionally, mutations in TP53, TTN, MUC16, MUC4, and HMCN1 were found to occur significantly more frequently in the high-risk group, whereas PIK3CA mutations were less common in the high-risk group compared to the low-risk group (**Supplementary Figure S11D**). The differences in gene mutations between the high-risk and low-risk groups were depicted in **Supplementary Figure S11E**, which demonstrated a higher mutation frequency in the high-risk cohort.

### The relationship between risk score and tumor microenvironment

**Figure 6:**
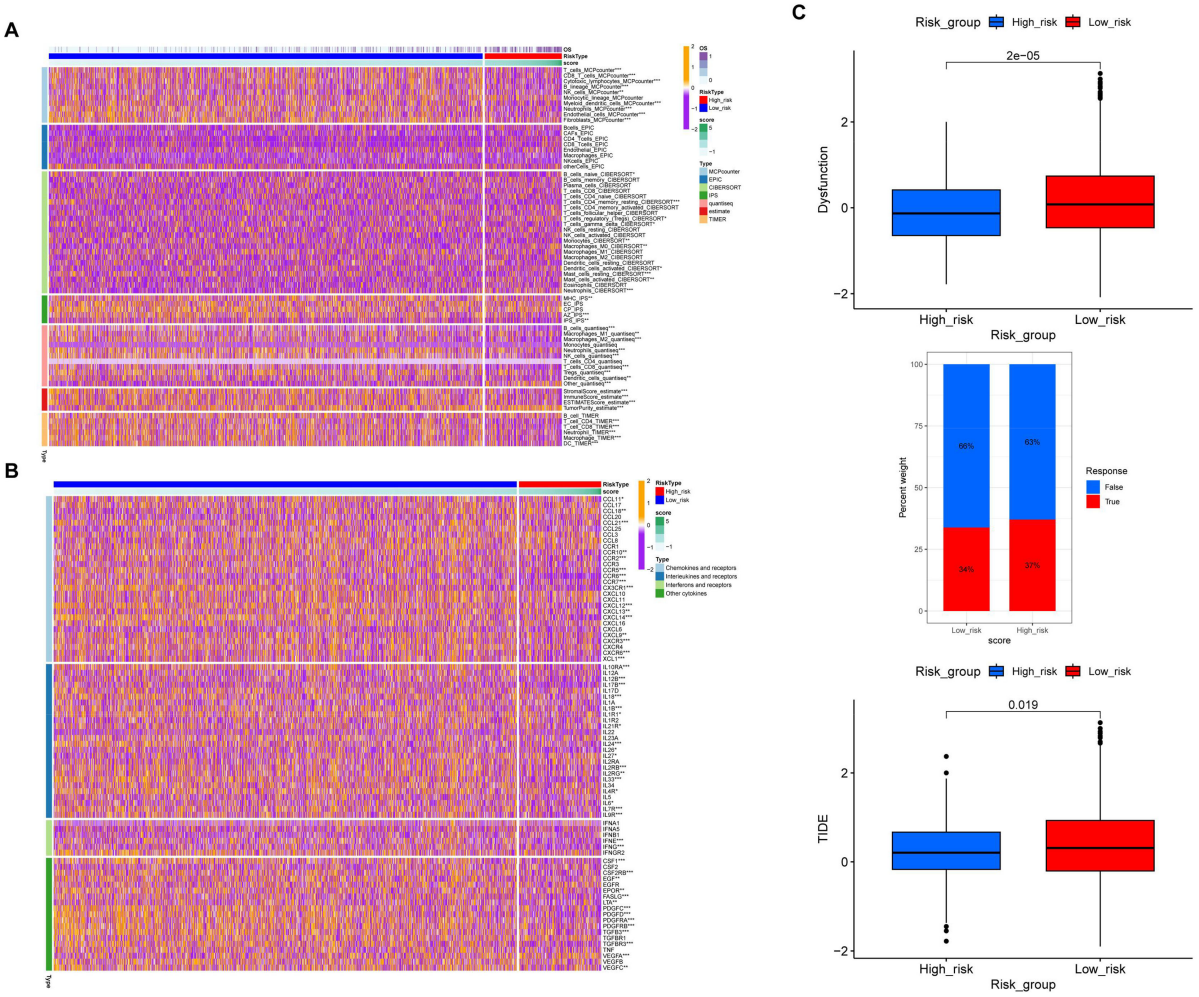
Immune correlation analysis of different lactylation risk scores in breast cancer. **(A)** The comparison of immune infiltration between the high- and low-score groups was presented. **(B)** The comparison of chemokine levels across the high- and low-score groups was provided. **(C)** The TIDE database was used to predict the TIDE score of patients and assess the efficacy of immunotherapy. The upper panel demonstrated the comparison of immune dysfunction between the high- and low-score groups. The middle panel showed the comparison of the percentage of responders to immunotherapy within the risk groups. The lower panel exhibited the distribution of TIDE scores in the high- and low-score groups.

As a crucial component of the tumor microenvironment (TME), the distribution of immune cells significantly influences immunotherapy strategies for patients with breast cancer. Therefore, we investigated the correlation between the risk model and the level of immune cell infiltration using the MCPCOUNTER, EPIC, CIBERSORT, IPS, QUANTISEQ, ESTIMATE, and TIMER algorithms. It was observed that the low-risk group showed markedly higher levels of immune cell infiltration, including B cells, CD4^+^ T cells, CD8^+^ T cells, and NK cells, in comparison to the high-risk group. The ESTIMATE algorithm was employed to assess tumor immune microenvironments, revealing that the low-risk group had higher ESTIMATE, immune, and stromal scores, along with lower tumor purity (**Figure 6A**). Furthermore, the relationship between the risk score and chemokine signatures was illustrated in **Figure 6B**. The TIDE database was utilized to predict the likelihood of immunotherapeutic response in breast cancer patients. A significant difference in the response rates to immunotherapy was observed between the two groups. The analysis indicated that the immune dysfunction score and TIDE score were higher in the low-risk group than in the high-risk group. In contrast, the high-risk group demonstrated a greater response rate to immunotherapy, indicating that this group may be less susceptible to tumor immune escape and that patients within this group may exhibit greater sensitivity to immunotherapy (**Figure 6C**).

### Correlation of the risk model with chemosensitivity and immunotherapy responses

**Figure 7:**
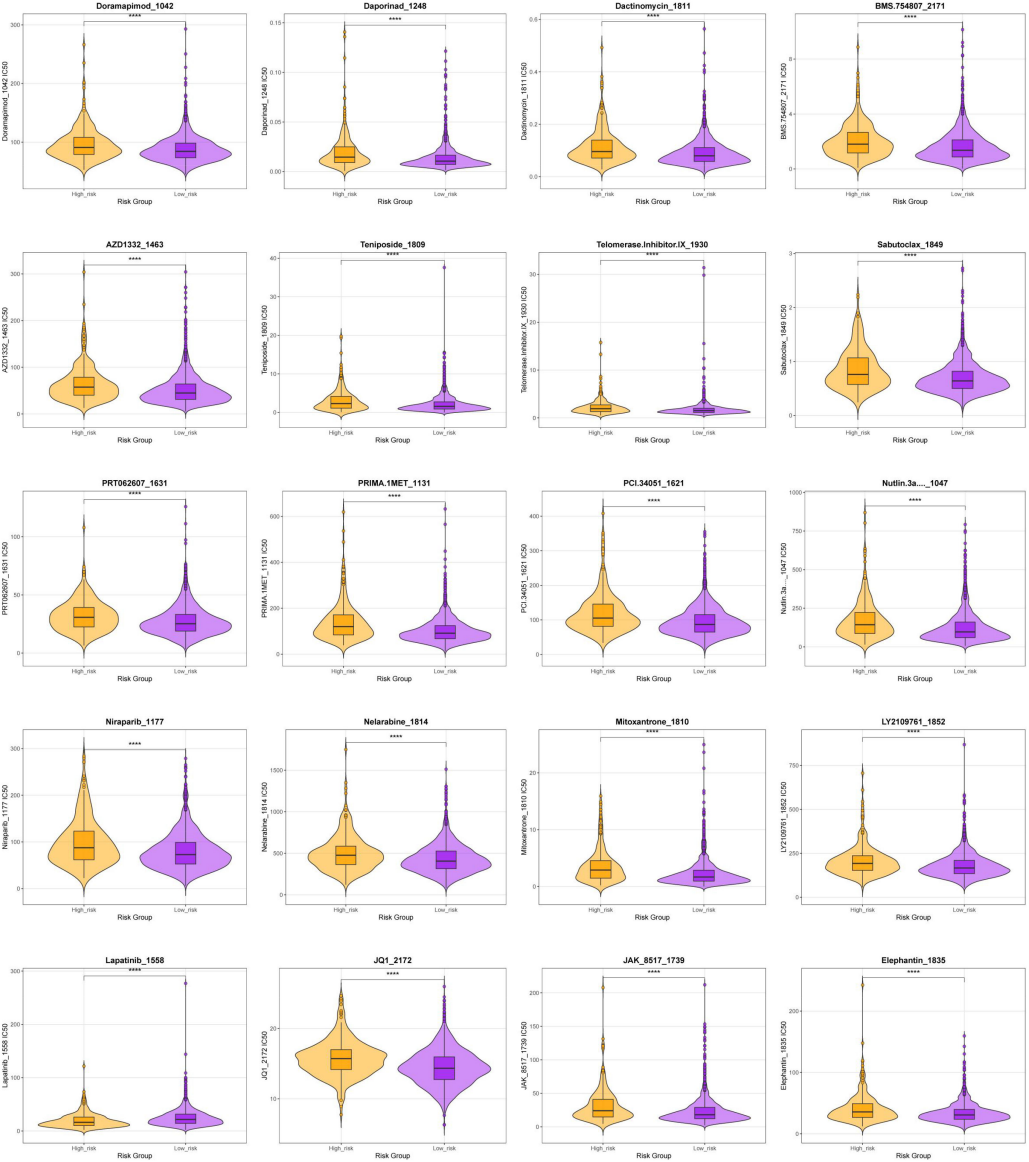
Drug sensitivity analysis among subgroups of a lactylation risk model for breast cancer. The IC50 values for various anticancer drugs were analyzed, revealing differences between the high- and low-score groups. Higher IC50 values indicated reduced sensitivity to treatment.

The chemotherapeutic drug responses of patients in both groups to conventional and innovative anticancer agents were assessed. The low-risk group exhibited greater sensitivity to several agents, including Doramapimod_1042, Daporinad_1248, Dactinomycin_1811, BMS-754807_2171, AZD1332_1463, Teniposide_1809, Telomerase Inhibitor IX_1930, Sabutoclax_1849, PRT062607_1631, PRIMA-1MET_1131, PCI-34051_1621, Nutlin-3a (-)_1047, Niraparib_1177, Nelarabine_1814, Mitoxantrone_1810, LY2109761_1852, JQ1_2172, JAK_8517_1739, and Elephantin_1835. In contrast, a higher responsiveness to Lapatinib_1558 was observed in the high-risk group (**Figure 7**). These findings indicate that the risk score may serve as a predictor of sensitivity to anticancer therapy.

### SUSD3 was significantly positively associated with fibroblasts in breast cancer

**Figure 8:**
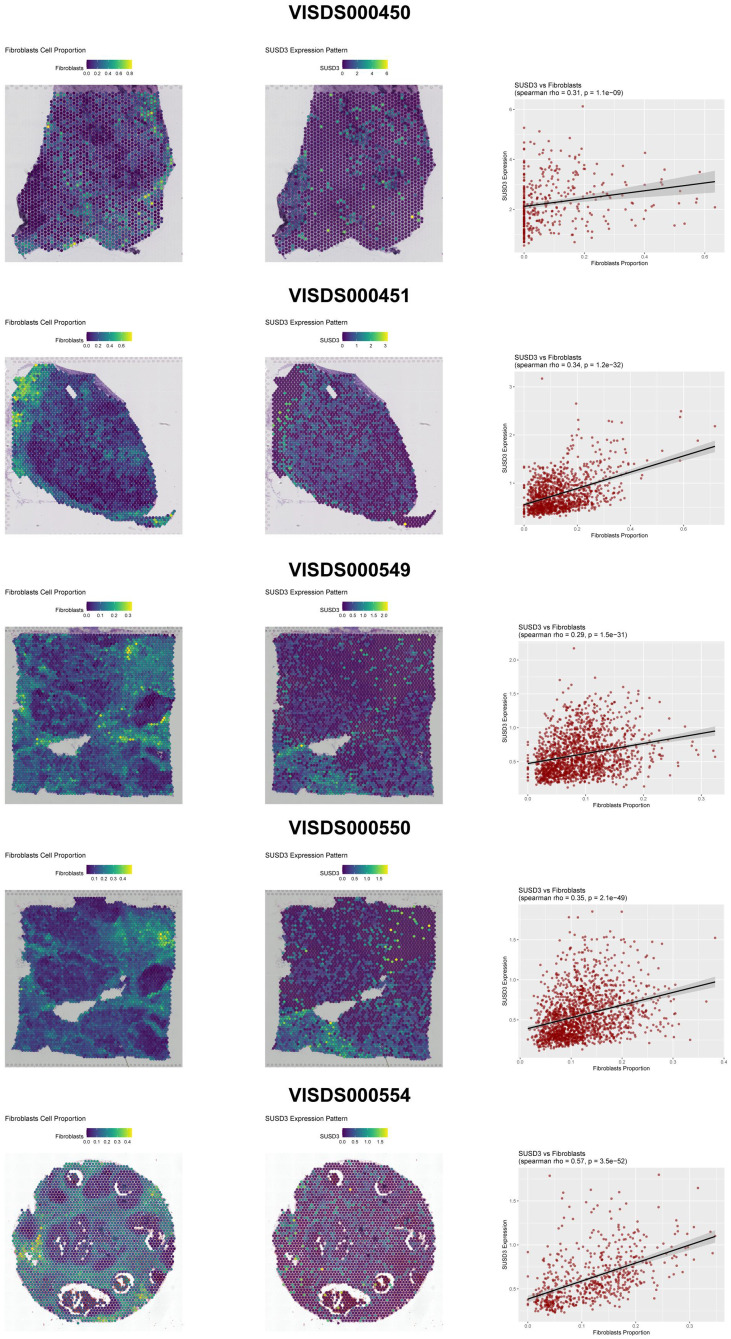
Spatial transcriptomics overview of SUSD3 in 5 breast cancer samples. The left panels showed the spatial distribution of fibroblasts across the 5 samples. The middle panels exhibited the spatial expression of SUSD3 in these samples. The right panels presented the positive correlation between SUSD3 expression and fibroblast presence across the 5 samples. .

Given the crucial roles of SUSD3 in the decision-making process of the model and its predictive accuracy, which underscores its potential as a biomarker for diagnostic and therapeutic applications, we conducted an exploration of the spatial distribution of SUSD3 in breast cancer using spatial transcriptomics analysis. It was observed that SUSD3 was distinctly positively correlated with fibroblasts (**Figure 8**), indicating that the elevated expression of SUSD3 in fibroblasts may significantly influence the progression and prognosis of breast cancer by regulating lactylation modification.

### Experimental validation of hub LRGs

**Figure 9:**
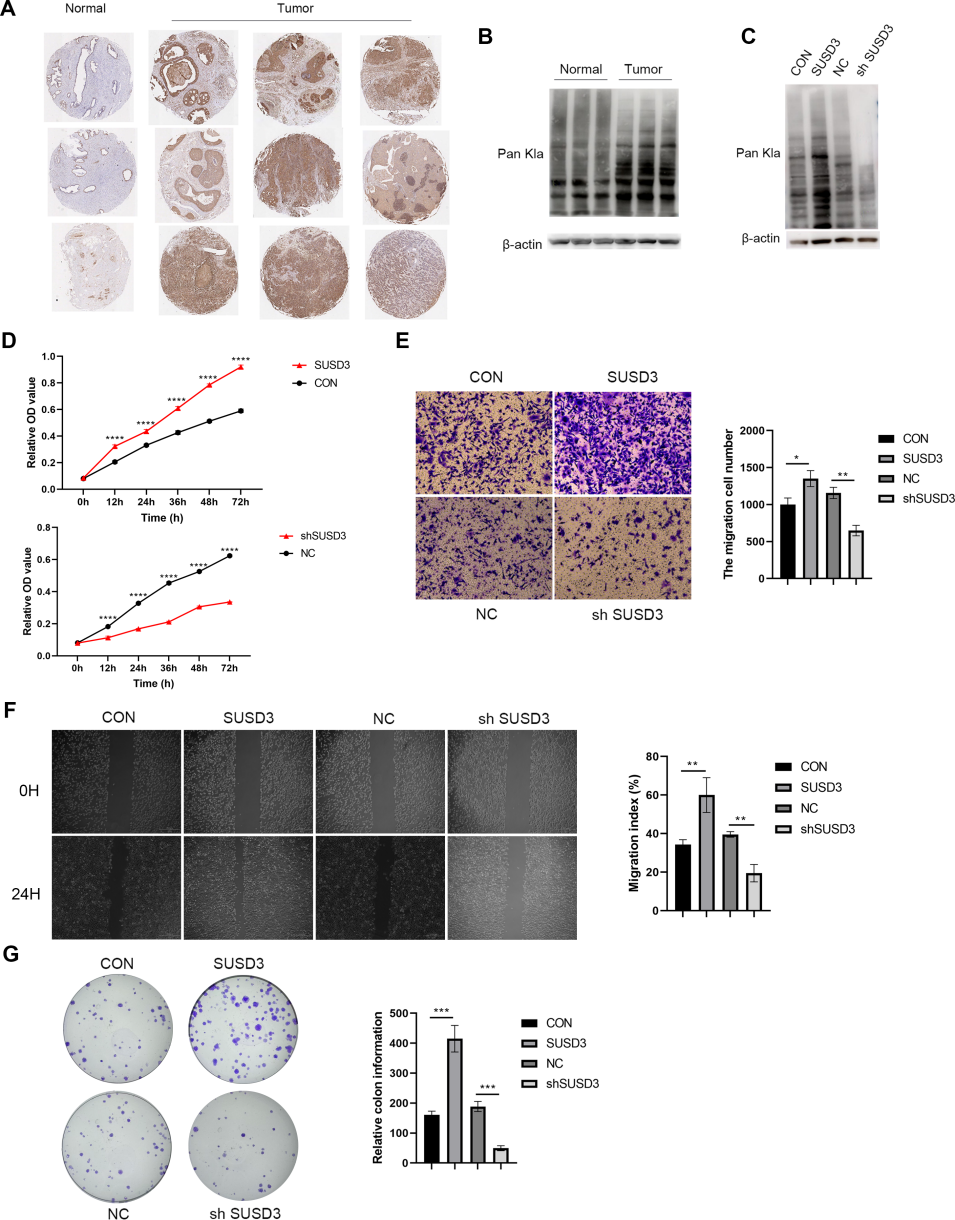
Experimental validation of hub LRGs. **(A)** The expression levels of SUSD3, G6PD, and FBXL6 in breast cancer tissues. **(B)** The different lactylation levels in breast cancer tissues and control tissues **(C)** The lactylation levels of MCF-7 cells when SUSD3 was overexpressed or downregulated. **(D)** The CCK-8 assay of MCF-7 cells when SUSD3 was overexpressed or downregulated. ****p ≤ 0.0001. **(E)** The migrated ability of MCF-7 cells when SUSD3 was overexpressed or downregulated. *p < 05, **p ≤ 0.01. **(F)** The wound healing assay of MCF-7 cells when SUSD3 was overexpressed or downregulated. **p ≤ 0.01. **(G)** The colony formation assay of MCF-7 cells when SUSD3 was overexpressed or downregulated. ***p ≤ 0.001.

Experimental validation was conducted using immunohistochemistry (IHC) on clinical breast samples to confirm the differential expression and potential roles of SUSD3, G6PD, and FBXL6 in breast cancer pathogenesis. As shown in **Figure 9A**, we observed significantly increased expression levels of SUSD3, G6PD, and FBXL6 in tumor tissues compared to normal tissues. Subsequently, we investigated the global lactylation levels in human breast cancer tissues relative to control tissues. We found that breast cancer tissues exhibited significantly elevated lactylation levels compared to control tissues (**Figure 9B**). Then we selected SUSD3 for further exploration of its influence on lactylation modification and found that SUSD3 was markedly positively correlated with the lactylation levels (**Figure 9C**). Finally, the results of the CCK-8, migration, wound healing, and colony formation assays demonstrated that SUSD3 significantly enhanced the proliferation and migratory abilities of breast cancer cells (**Figures 9D–G**).

## Discussion

As a crucial post-translational regulatory mechanism, lactylation has garnered significant attention due to its role in cancer progression (6, 12, 30, 31). Lactylation can occur in both histone and non-histone proteins. In histones, lactylation regulates gene transcription through epigenetic mechanisms. However, the identity of the readers for histone lactylation remains unclear, despite the identification of the corresponding writers and erasers. Most studies have focus on specific gene sets, leaving the question of whether histone lactylation positively or negatively regulates gene expression unresolved (6, 32). In contrast, research on non-histone proteins has demonstrated that lactylation can enhance or suppress their functions (23, 33–35). Whether lactylation can confer entirely new functions to these proteins, however, remains unknown.

Numerous studies have elucidated the molecular mechanisms linking lactylation to cancer progression, including cell proliferation, metastasis, metabolism, and chemotherapy resistance (10, 17, 30, 36). These findings suggest that targeting lactylation could represent a promising strategy for cancer diagnostics and treatment, although many regulatory mechanisms remain to be fully understood. In comparison to other PTMs such as acetylation, phosphorylation, and ubiquitination, the specific role of lactylation in breast cancer remains largely unexplored, highlighting the need for further investigation into its therapeutic implications.

Multi-omics analysis integrates data from various genetic levels, including transcriptomics, genomics, and metabolomics, to comprehensively investigate tumor characteristics and the influences of lactylation on tumors. This approach enhances our understanding of the molecular mechanisms underlying tumors and contributes to the discovery of new biomarkers and drug targets, which may facilitate the development of predictive, preventive, and personalized medicine (37).

In the current study, distinct cell types within breast cancer were identified using scRNA-seq, highlighting the complexity of the TME. Notably, the assessment of lactylation scores across these cell types exhibited significant variability.

Furthermore, a comprehensive multi-omics analysis was conducted on publicly available data to evaluate the lactylation phenotype of breast cancer. This analysis aimed to clarify the differences in gene expression, survival prognosis, immune infiltration levels, functional enrichment, genomic mutations, stemness features, chemotherapeutic drug responses, and clinicopathological characteristics across different lactylation phenotypes. A LAGPI model was developed using 10 classical algorithms combined with 99 machine-learning algorithm combinations, with the RSF algorithm selected due to its highest C-index, demonstrating exceptional accuracy in predicting breast cancer prognosis in both training and test datasets. This underscores its potential for future clinical applications.

Based on the LAGPI, patients with breast cancer were stratified into high- and low-LAGPI groups, which were identified as independent prognostic risk factors through univariate and multivariate regression analyses. The high-risk group was associated with shorter survival times and worse prognoses, leading to adverse clinical outcomes. Subsequently, the LAGPI was integrated with multiple clinical features (TNM stage, grade, and OS) to construct a tumor predictive nomogram. The LAGPI exhibited effective prognostic prediction capabilities and clinical utility, potentially aiding in the timely identification of patients with poor prognosis for breast cancer and facilitating the formulation of early and targeted interventions to improve patient outcomes.

Immunotherapy has significantly altered the treatment landscape for breast cancer by targeting the signaling pathways that facilitate tumor evasion of immune surveillance. Nonetheless, not all patients derive sustained benefits from immunotherapy, emphasizing the necessary for precise identification of individuals who are more likely to respond to these treatments (1, 38). While it is recognized that lactate plays a role in drug resistance by influencing cellular metabolism and the acidification of TME, the specific contribution of lactylation to this process remains inadequately understood. In our study, we utilized 7 algorithms to estimate immune cell infiltration between high- and low-risk groups, revealing distinct immune responses and clinical outcomes for both groups. These results highlight the crucial role of LRGs in chemoresistance, and suggest that the LAGPI model may serve as a valuable tool for identifying breast cancer patients who are more responsive to immunotherapy, thereby enhancing personalized treatment strategies. Recent research indicated that metabolic reprogramming, including lactate accumulation, may contribute to the failure of cancer therapies; however, the underlying mechanisms require further investigation.

To improve the predictive accuracy of immunotherapy outcomes, it is crucial to incorporate multiple biomarkers, given the complexity of the immune system. TMB has been found to correlate with T cell infiltration, tumor neoantigen burden, and response to immune checkpoint inhibitors (ICIs) across various solid tumor types (39). Moreover, TMB serves as a predictive marker for immunogenicity, with elevated TMB levels leading to the production of more neoantigens, which correlates with a more favorable response to immunotherapy (40, 41). Our investigation revealed that patients in the high-risk group showed a higher number of mutations compared to those in the low-risk group; these additional mutations were linked to increased sensitivity to immunotherapy. Furthermore, we observed a negative correlation between the TIDE score and the LAGPI, signifying a reduced likelihood of tumor immune escape in the high-risk group. These findings suggest that the LAGPI may enhance the development of personalized immunotherapy strategies for breast cancer patients in the future.

Through RSF regression and SHAP analysis, SUSD3 was identified as the gene having the most significant impact on the predictive accuracy of our model. SUSD3 (Sushi Domain-Containing 3) is a cell surface protein characterized by extracellular, transmembrane, and cytoplasmic domains, with notably high expression in estrogen-sensitive tissues, particularly in breast cancer. Previous studies has reported that SUSD3 is involved in estrogen-dependent metastatic processes and serves as a potential biomarker for predicting both the occurrence and prognosis of breast cancer (42). To further investigate the spatial distribution of SUSD3 in breast cancer, we conducted spatial transcriptomics analysis, which revealed a notable positive correlation between SUSD3 expression and fibroblasts. This finding suggests that the elevated SUSD3 levels in fibroblasts may influence the progression and prognosis of breast cancer through the modulation of lactylation, a relationship that has not been previously reported.

The LAGPI performed in this study identified key immune-related features and their correlations with patient prognosis, suggesting that LRGs not only function as biomarkers in clinical settings but also play an indispensable role in immune modulation and chemoresistance. Additionally, we observed higher levels of lactylation, as well as increased expression of SUSD, G6PD, and FBXL6 in breast cancer tissues compared to normal tissues. Furthermore, our findings demonstrated that SUSD3 promotes the proliferation and migration of breast cancer cells, highlighting its potential role in tumor progression.

The lactylation-related genes selected for model construction have been associated with distinct biological processes relevant to cancer progression. For instance, G6PD modulates NADPH levels, which in turn influence cellular proliferation, survival, and stress responses; however, its enzyme activity is inhibited by lactylation (43). Additionally, lactylation of H2BK58 mediated by LDHA regulates the expression of NDRG1 (44). In turn, NDRG1 stabilizes LDHA, enhancing glycolysis, lactate accumulation, and promoting H3K18 lactylation (45). ELOVL1 may influence tumor cell proliferation by regulating the activation, proliferation, and metabolic reprogramming of CD8^+^ T cells (46). FBXL6 has been shown to activate aerobic glycolysis, thereby contributing to tumor malignancy (47). Zhao et al. identified the lactate-DHCR7 axis as a crucial biomarker involved in cisplatin resistance and its impact on the efficacy of immunotherapy in bladder cancer (48). Although previous studies have examined the relationship between lactylation signatures and breast cancer prognosis (49–51), our analysis using the lactylation-associated gene panel (LAGPI) identified new biomarkers alongside DHCR7 and G6PD. Notably, direct evidence linking SUSD3, HGH1, SLC52A2, and THEM6 specifically to lactylation modifications in breast cancer remains lacking. Furthermore, we conducted *in vitro* experiments that confirmed the role of SUSD3 in promoting the malignant phenotype of breast cancer cells, providing experimental evidence to support SUSD3 as a potential therapeutic target.

However, several limitations exist in this study. First, further experimental validation, both *in vitro* and *in vivo*, is needed to provide more robust support for the conclusions drawn. Second, the reasons behind the observed phenomenon in the high-risk group- characterized by a lower TIDE score and higher TMB, yet exhibiting lower immune infiltration and poorwe prognosis compared to the low-risk group-necessitate further investigation. Finally, multicenter, large-scale studies are essential to validate the prognostic significance of the LAGPI.

In summary, we identified 14 LRGs that are correlated with the prognosis of breast cancer patients. By integrating these genes, we developed the LAGPI using multiple machine learning algorithms, which demonstrated significant predictive capabilities for distinguishing patient outcomes and response to immunotherapy. This suggests that targeting lactylation may present new therapeutic opportunities. Future research should focus on exploring the functional mechanisms of lactylation within the TME to fully assess its potential as a therapeutic target in breast cancer. We anticipate that the well-organized data presented in this study will facilitate future investigations into the underlying mechanisms of lactylation modification in breast cancer.

## Data Availability

The original contributions presented in the study are included in the article/**Supplementary Material**. Further inquiries can be directed to the corresponding author.
