# Supplementary material for: Development and validation of an interpretable machine learning model identify the lactylation-related protein SUSD3 as a prognostic and therapeutic biomarker for breast cancer
Source: Front Immunol. 2026 Jan 27;17:1701978. doi: 10.3389/fimmu.2026.1701978 (PMC12886455; doi:10.3389/fimmu.2026.1701978)

**Figure S1. Heatmap of the correlation between lactylation score and HALLMARK pathway in each cell type**

Each cell was scored based on the HALLMARK pathway from the MsigDB database, and the correlation between the Lactylation score and the HALLMARK pathway score is shown

**
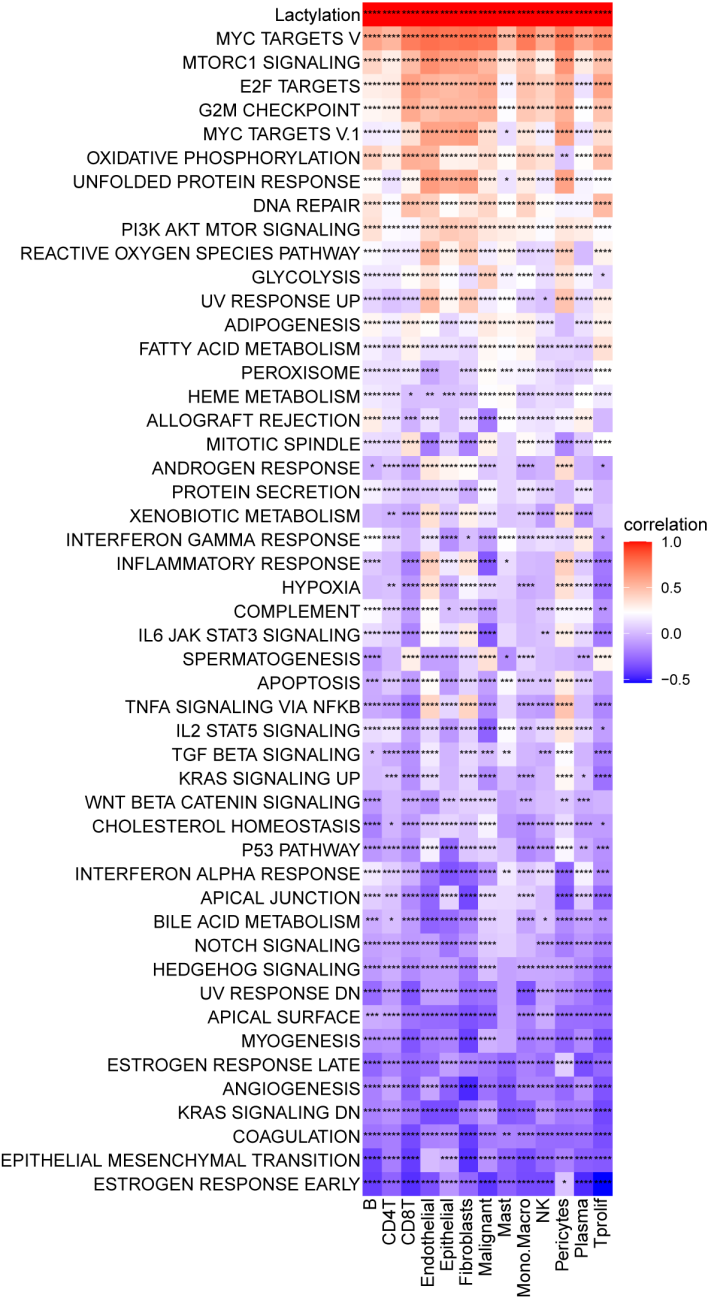
**

**Figure S2. Cell communications between the high- and low-score groups of lactylation gene set scoring**

**A.** Network diagrams demonstrated enhanced intercellular communication within the lactylation-high group

**B.** A bar graph exhibited cellular communication by displaying both the number and strength of interactions

**C.** The number of signaling pathways was compared between the lactylation-high and lactylation-low groups

**D.** Signaling dynamics were compared between the lactylation-high and lactylation-low groups, proving more robust signaling in the lactylation-high group

**E.** Heatmaps showed the outgoing (left) and incoming (right) signal strength for each signaling pathway among different cell types

**
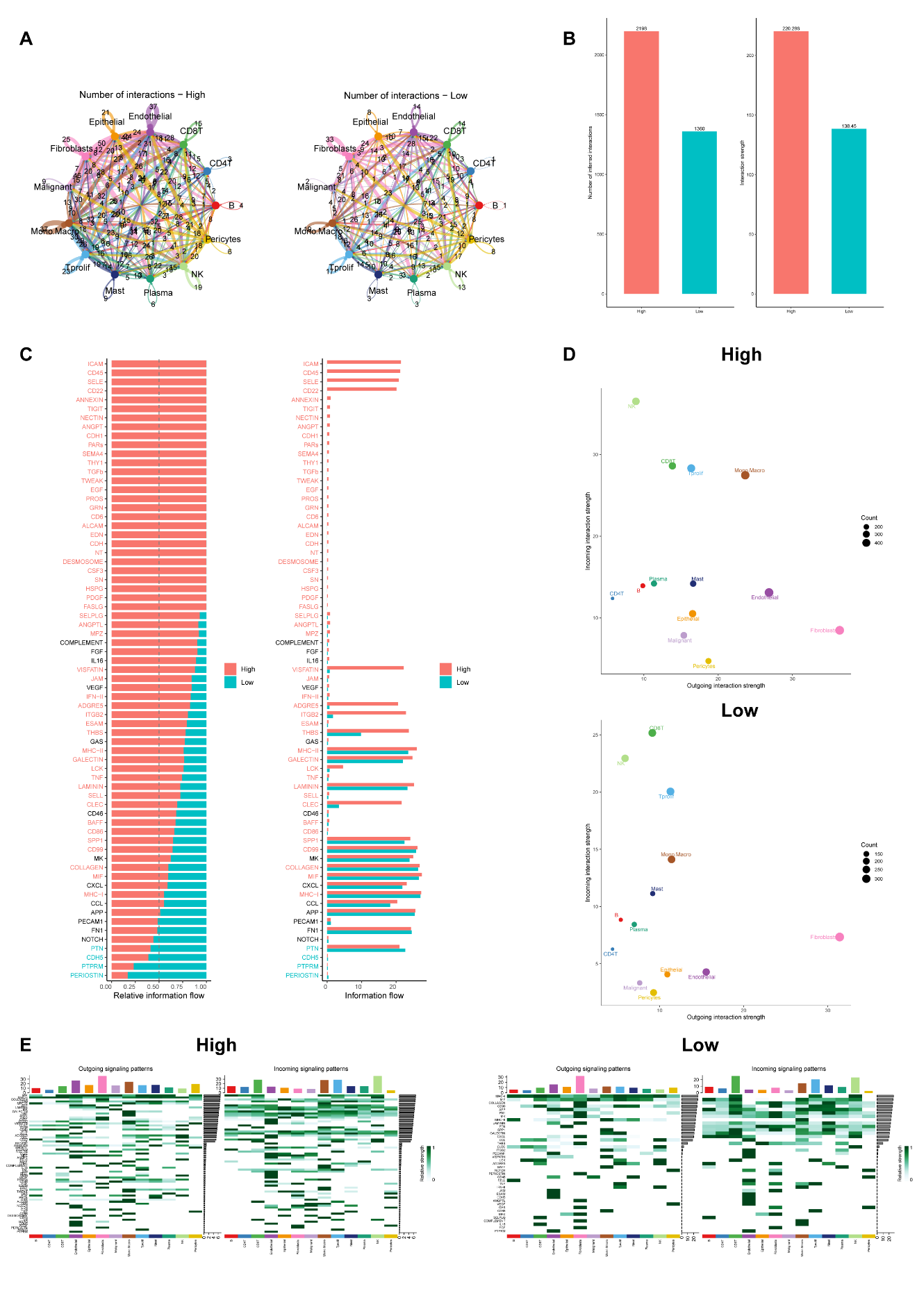
**

**Figure S3. Gene correlation analysis and univariate Cox regression analysis**

Gene correlation analysis, along with univariate Cox regression analysis, was used to identify 35 genes with significant prognostic value (p < 0.01) for further investigation. The purple sections on the right half of the circle indicate prognostic risk factors, while the green sections represent protective factors. The circle size corresponds to the p-value, and the presence of a connection indicates a correlation between genes where p<0.05

**
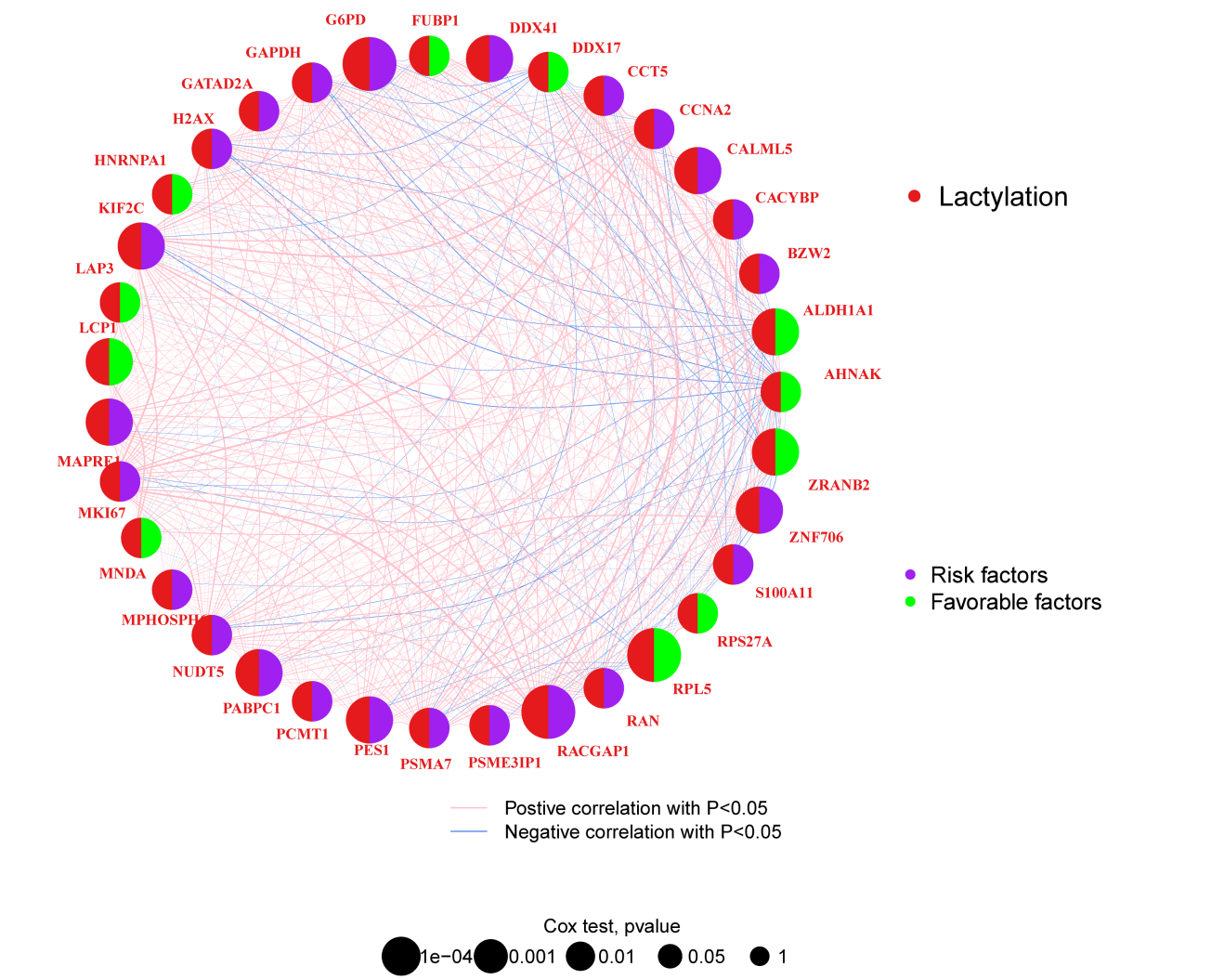
**

**Figure S4. Genetic alterations across the 3 clusters**

**A-C.** Waterfall plots showed the clusters of genes with the highest frequency of somatic mutations in **(A)** cluster A, **(B)** cluster B, and **(C)** cluster C

**D-F.** Differences in genetic mutations between the groups were presented

**
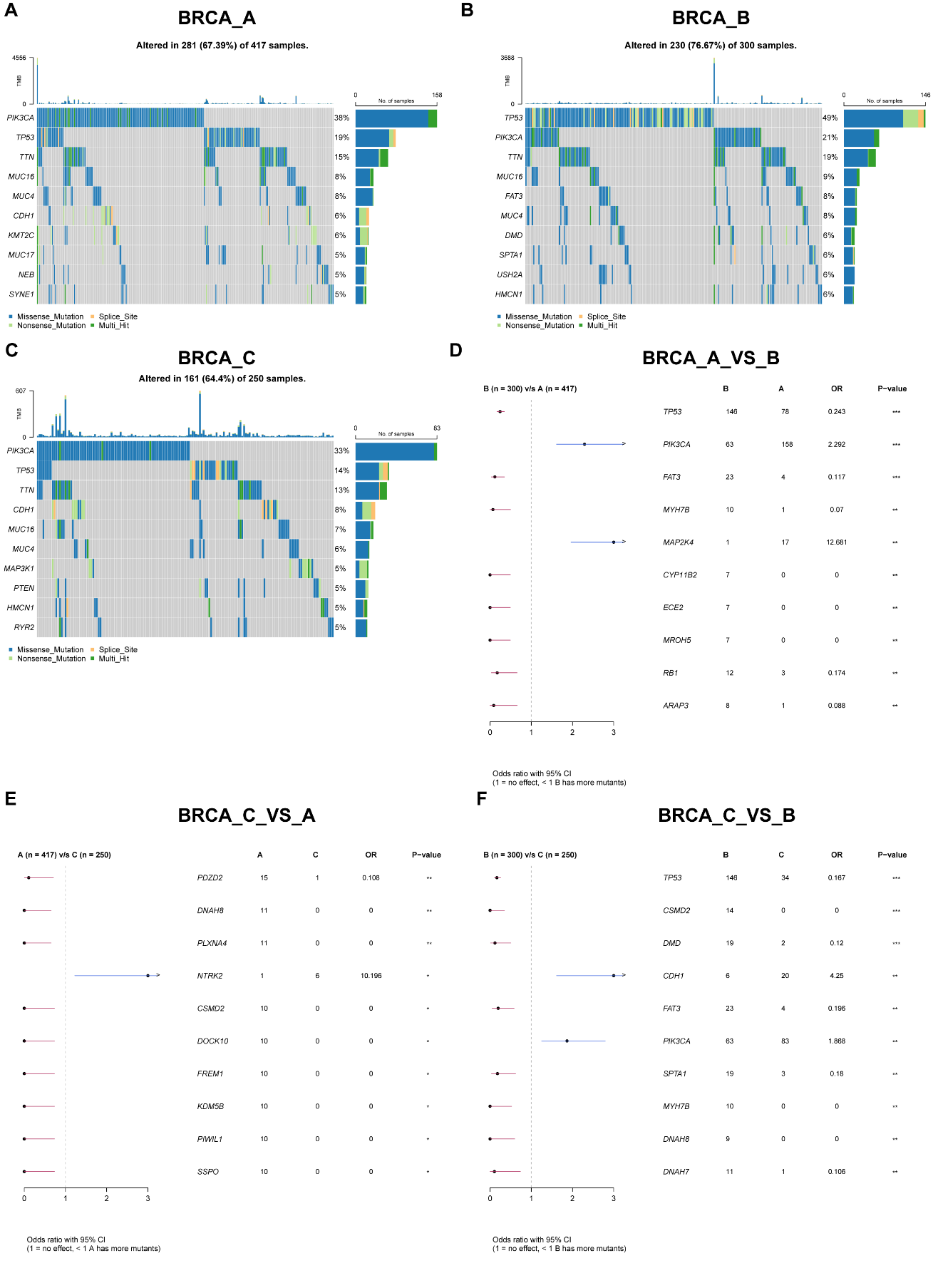
**

**Figure S5. The landscape of the correlation between lactylation typing and immune infiltration in breast cancer**

**A.** The comparison of immune infiltration across the 3 clusters was shown

**B.** A heatmap obviously clarified the variations in immune cell infiltration within the 3 clusters, as measured using 7 predictive algorithms. ns p > 0.05, *p < 0.05, **p < 0.01, ***p < 0.001

**
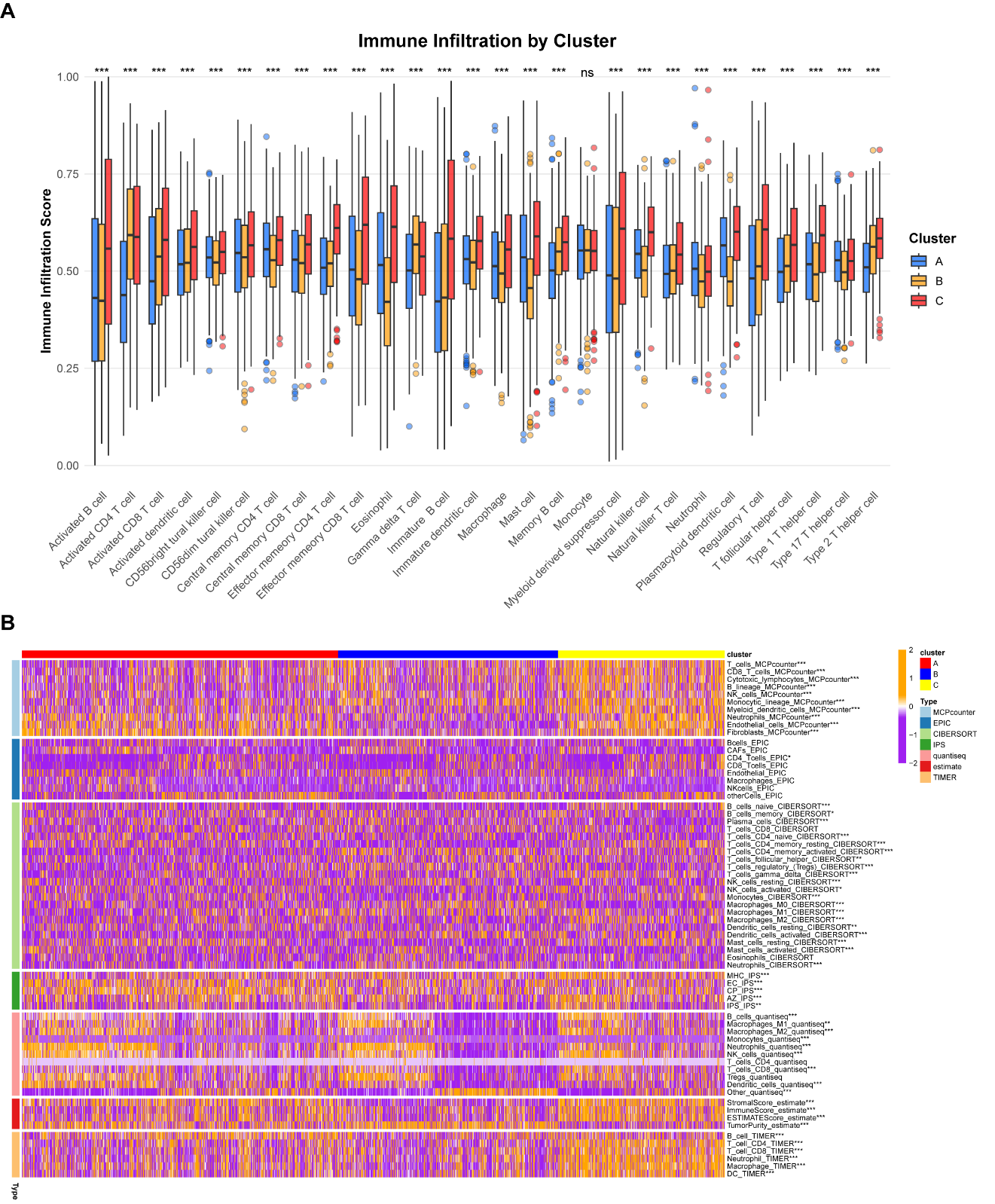
**

**Figure S6 Functional analysis based on DEGs among** **lactylation prognosis-associated clusters**

1. The top ten enriched Gene Ontology (GO) terms are categorized into Biological Process (BP), Cellular Component (CC), and Molecular Function (MF)
2. The top ten enriched KEGG pathways are presented

**
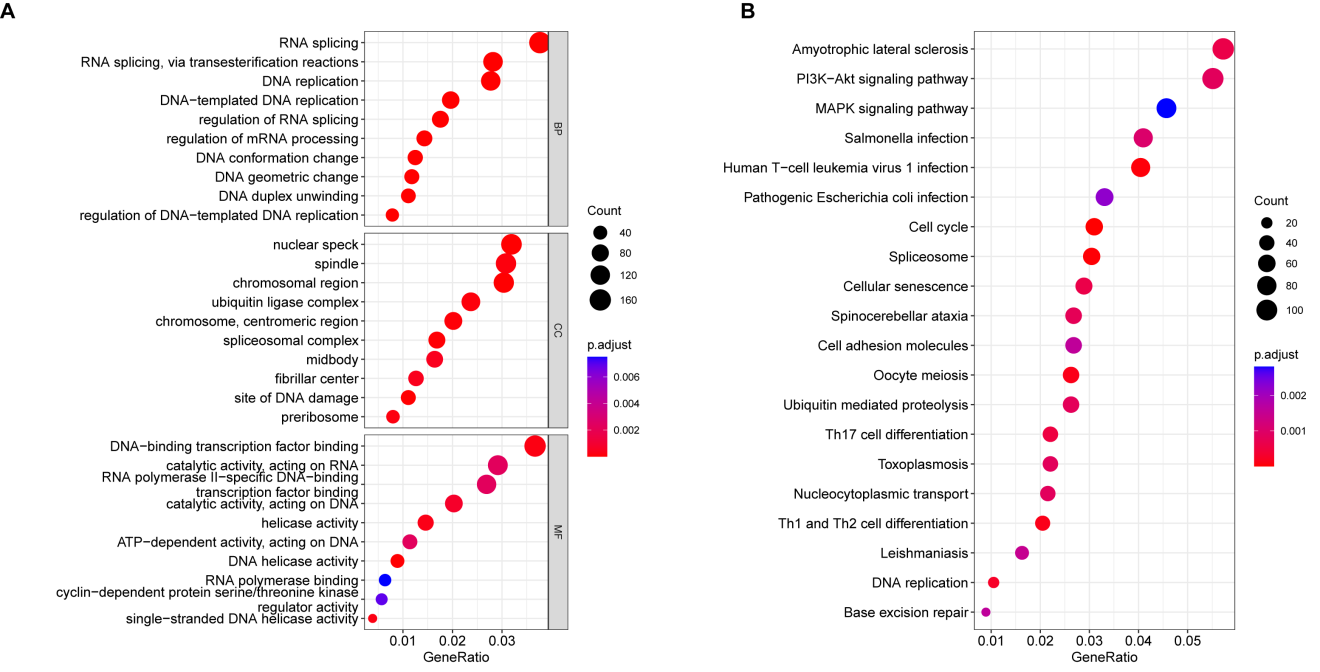
**

**Figure S7. Differentially expressed genes (DEGs) identified among the 3 clusters**

**A total of 5640 DEGs were identified, with a threshold for differential expression set at |logFC |>0.5, and adjusted p ＜0.05. Univariate regression analysis was performed on all 5640 subtype-related genes, revealing that 14 of these genes are significantly related to prognosis across at least 5 breast cancer datasets.**

**
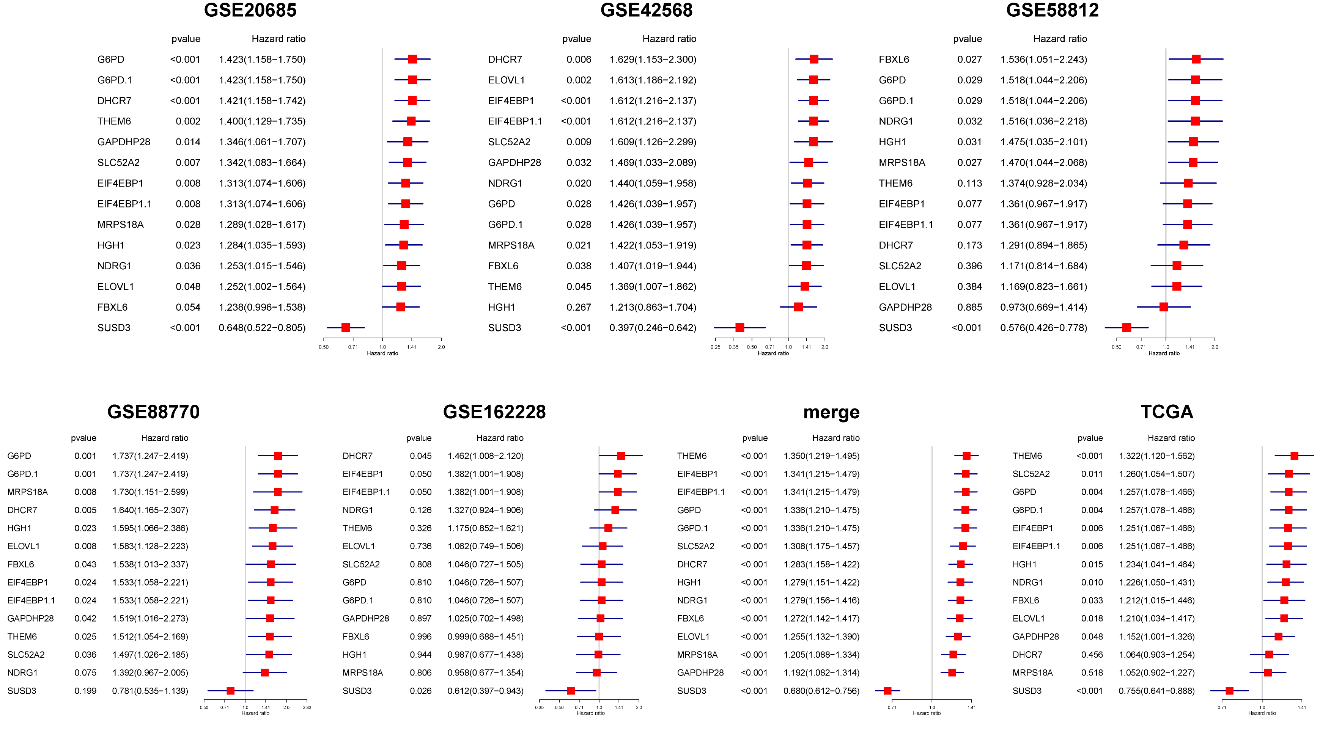
**

**Figure S8. The relationships between clinicopathological features and risk scores**

**A.** The left panel showed the variation in scores across different grade groups, while the right panel exhibited the distribution of grades within the high- and low-score groups

**B.** The left panel contrasted the scores among M-stage groups, and the right panel illustrated the distribution of M-stage between the high- and low-score groups

**C.** The left panel demonstrated the differences in scores across N-stage groups, whereas the right panel showed the distribution of N-stage between the high- and low-score groups

**D.** The left panel illustrated the difference in overall survival (OS) status between the high- and low-score groups, while the right panel showed the distribution of OS status within these groups

**E.** The left panel showed the variation in scores among different stage groups, whereas the right panel exhibited the distribution of stages between the high- and low-score groups

**F.** The left panel demonstrated the differences in scores among T-stage groups, while the right panel showed the distribution of T-stage between the high- and low-score groups

**
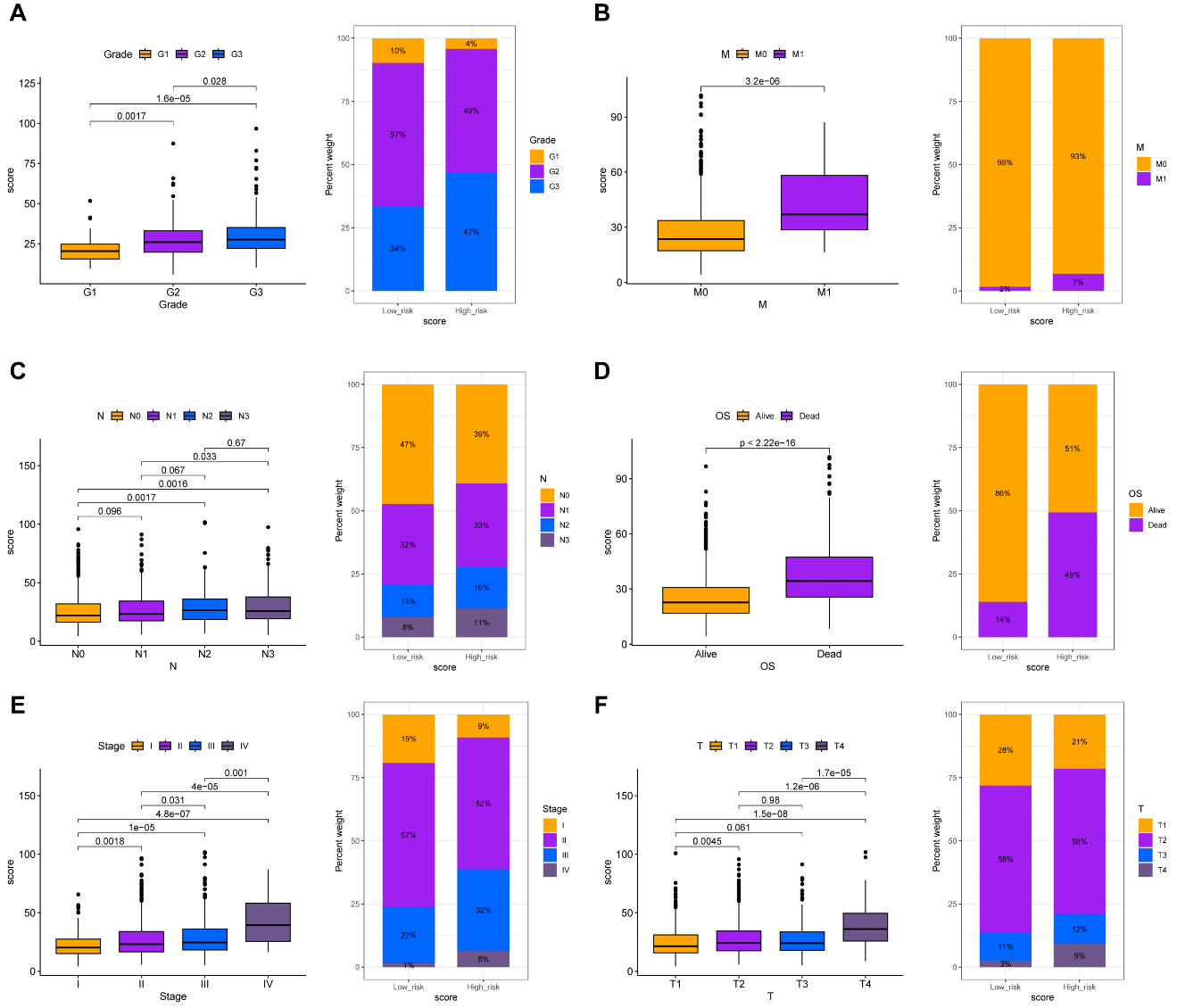
**

**Figure S9. Prognostic analysis of** **genes associated with lactylation risk score in breast cancer**

1. Univariate model analysis for prognostic models across different lactylation subtypes of breast cancer is displayed
2. Multifactorial model analysis for prognostic models across different lactylation subtypes of breast cancer is shown

**
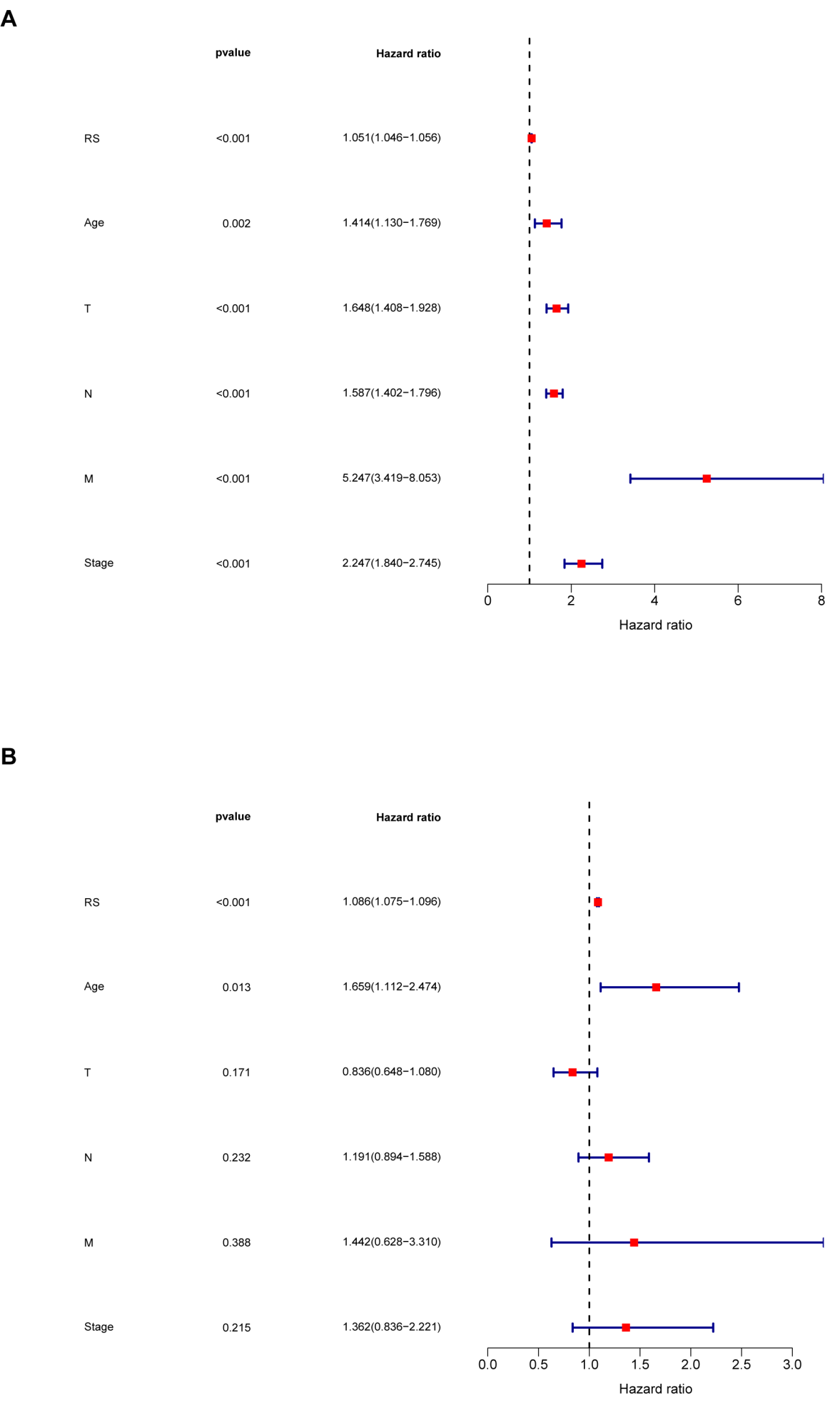
**

**Figure S10. Enrichment analysis of genes associated with** **lactylation risk score in breast cancer**

1. The top 50 genes positively associated with the lactylation risk score are listed
2. The top 50 genes negatively associated with lactylation risk score are identified
3. Functional enrichment analysis results of the positively correlated top 50 genes are shown, including Gene Ontology (GO), KEGG, and Reactome results for the correlated genes

**
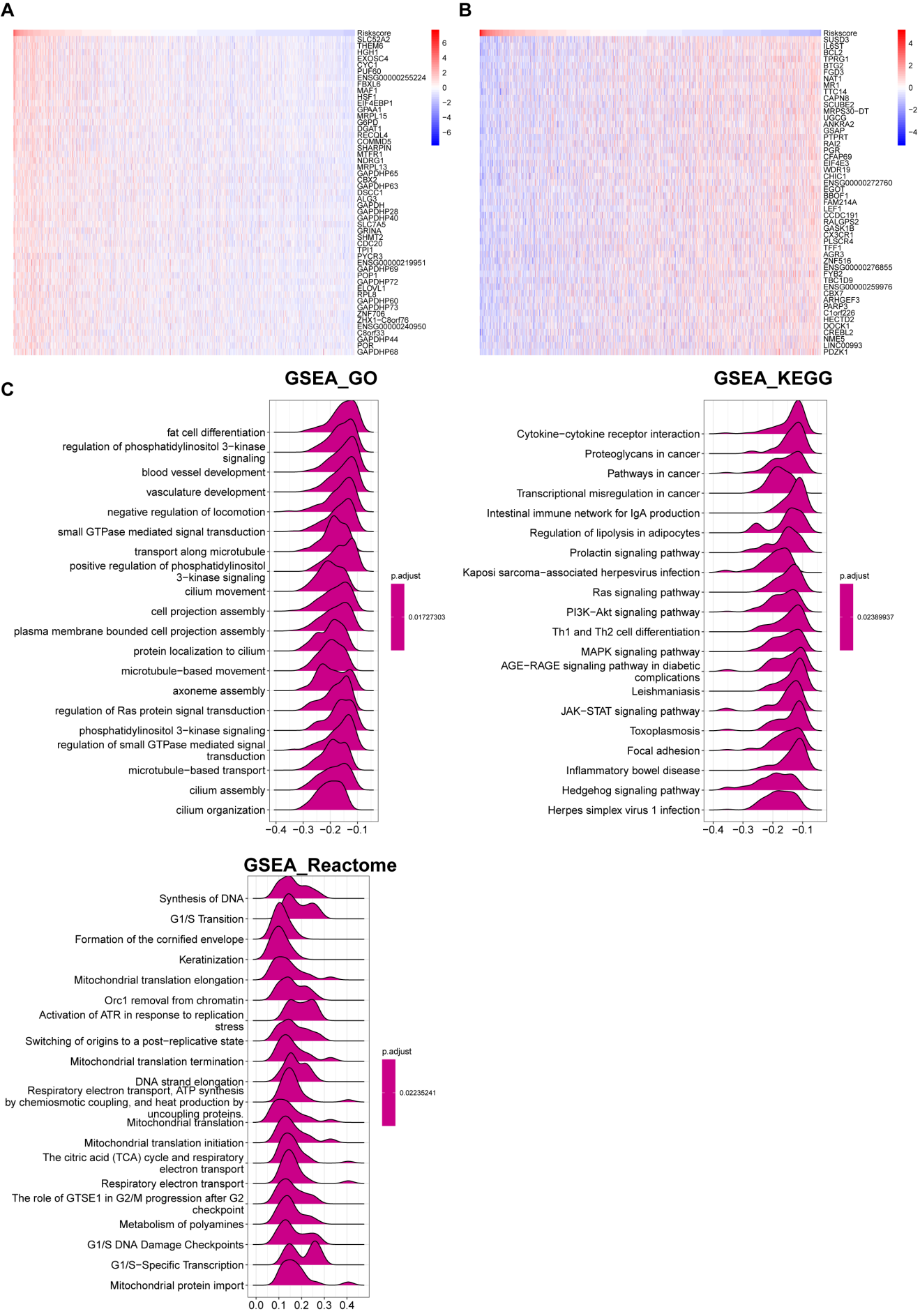
**

**Figure S11. Correlation of** **lactylation risk score with stemness**

1. mDNAsi serves as an indicator for measuring cell stemness at the DNA level. The left panel showed the diffring mDNAsi scores between the high- and low-risk groups. The middle panel exhibited the Pearson correlation analysis between breast cancer risk scores and mDNAsi. The right panel illustrated the differences in mDNAsi scores between the high- and low- risk groups
2. mRNAsi is employed as an indicator for assessing cell stemness at the mRNA level. The left panel showed the differing mRNAsi scores between the high- and low-risk groups. The middle panel exhibited the Pearson correlation analysis between breast cancer risk scores and mRNAsi. The right panel illustrated the differences in mRNAsi scores between the high- and low- risk groups
3. Stemness-associated signatures were collected, and ssGSEA analysis was performed, revealing the distribution of stemness scores across high- and low-risk groups
4. Waterfall plots were presented, showing clusters of genes exhibiting the highest frequency of somatic mutations in both the high-risk (upper) and low-risk (lower) groups
5. The differences in gene mutations between the high- and low-risk groups were highlighted


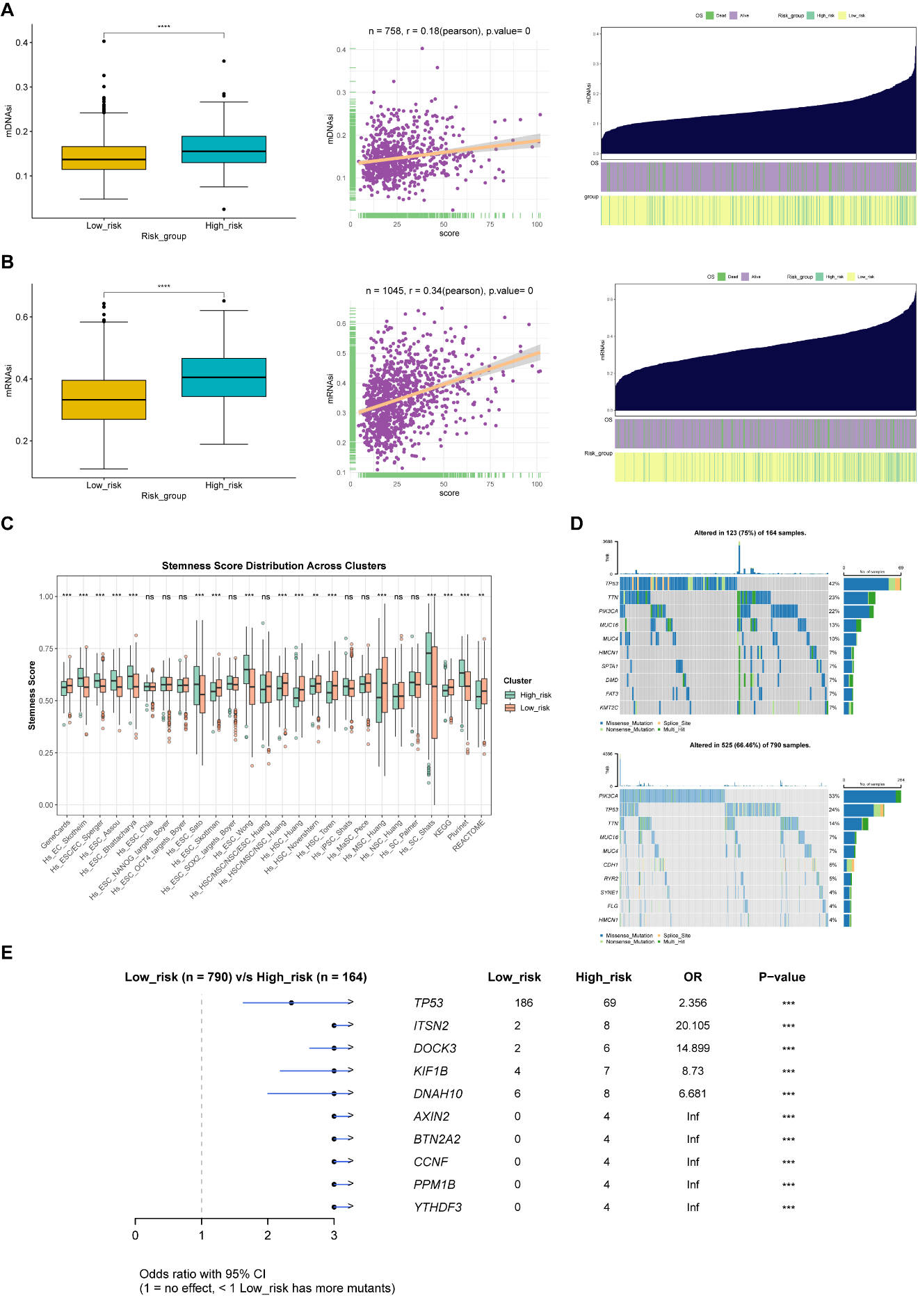

Supplement: Supplementary file 1 [file Table1.docx]
