# Supplementary figures and images for: Development and validation of an interpretable machine learning model identify the lactylation-related protein SUSD3 as a prognostic and therapeutic biomarker for breast cancer
Source: Front Immunol. 2026 Jan 27;17:1701978. doi: 10.3389/fimmu.2026.1701978 (PMC12886455; doi:10.3389/fimmu.2026.1701978)

Figure 9A


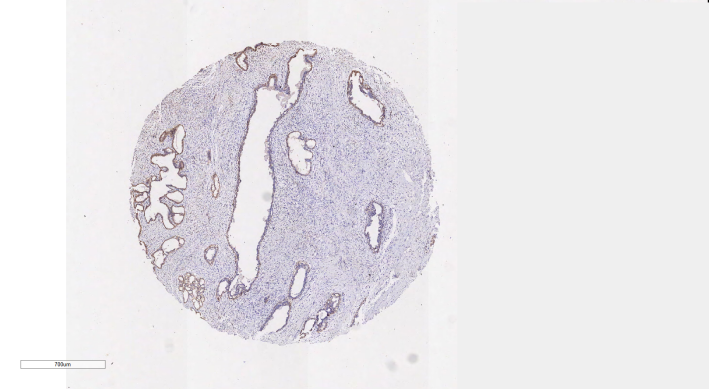

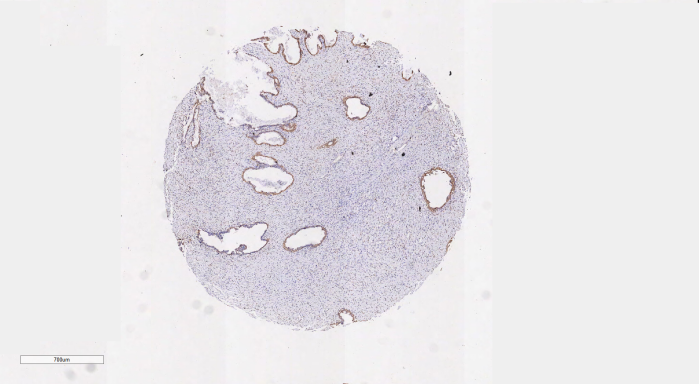

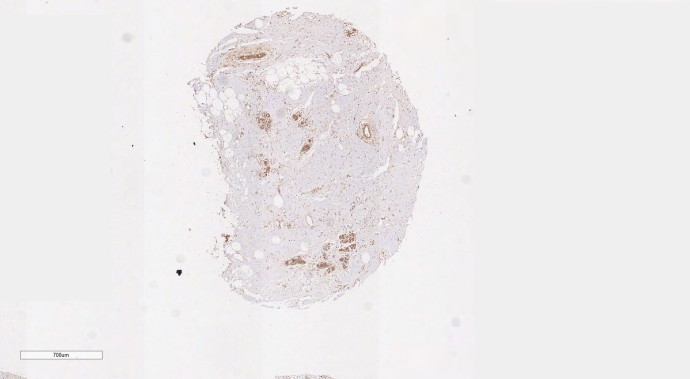


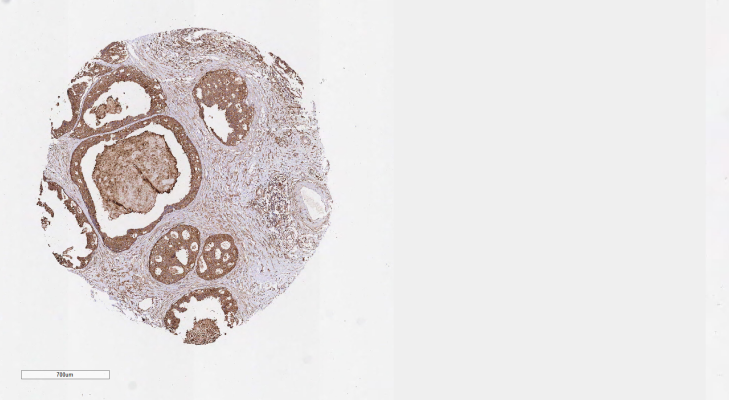

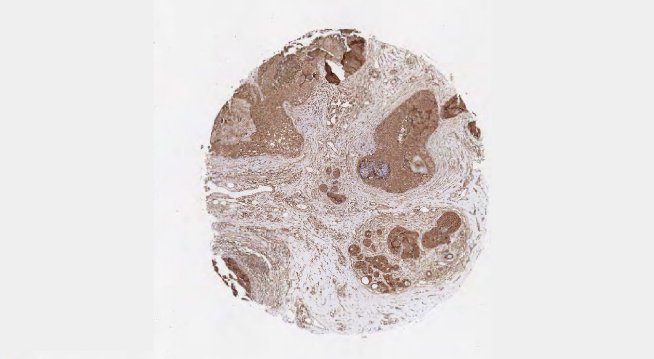

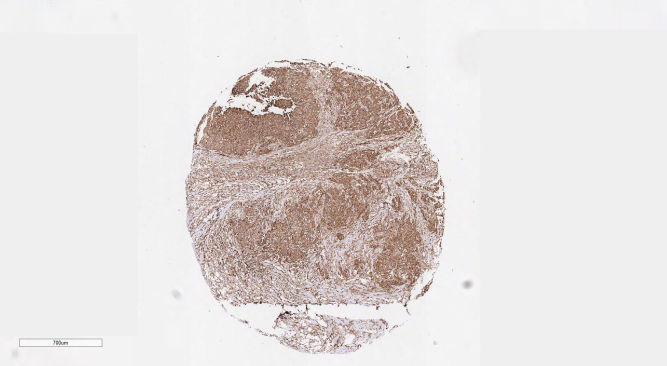


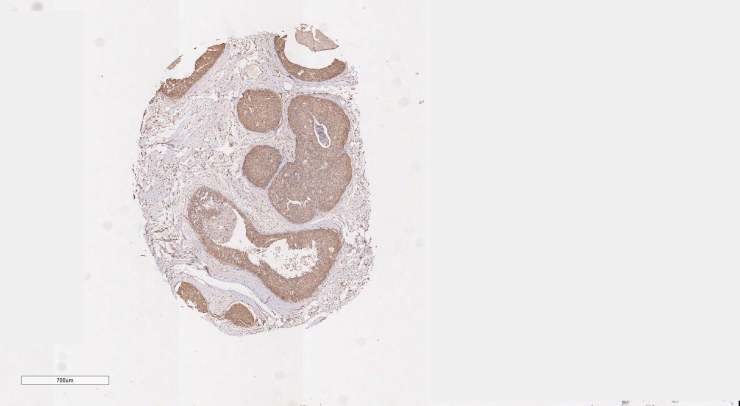

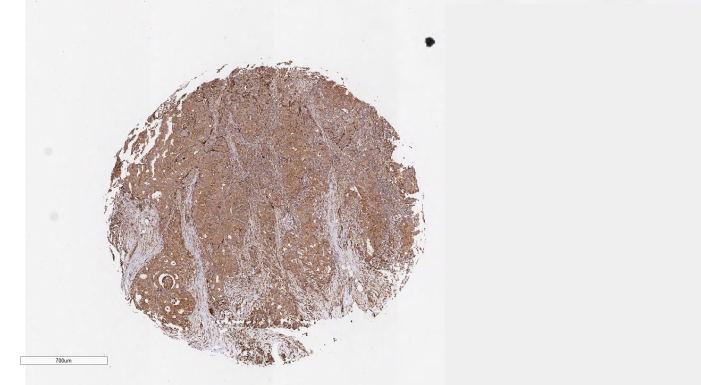

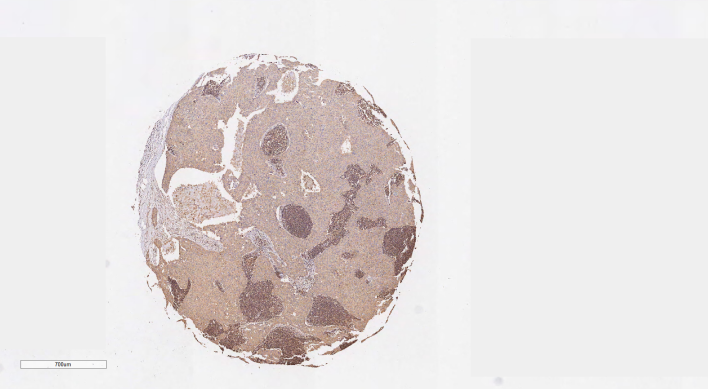


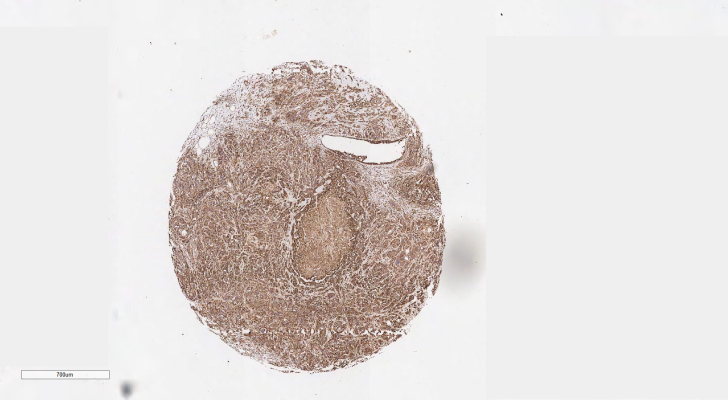

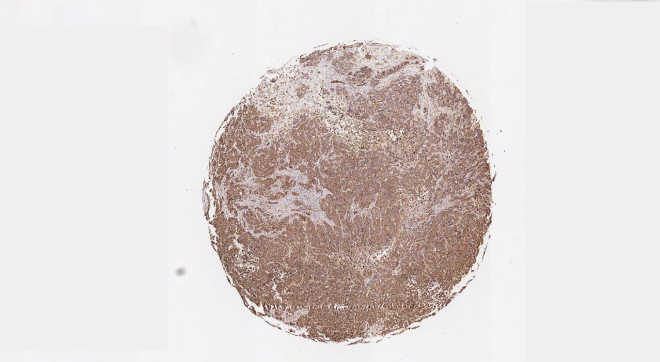

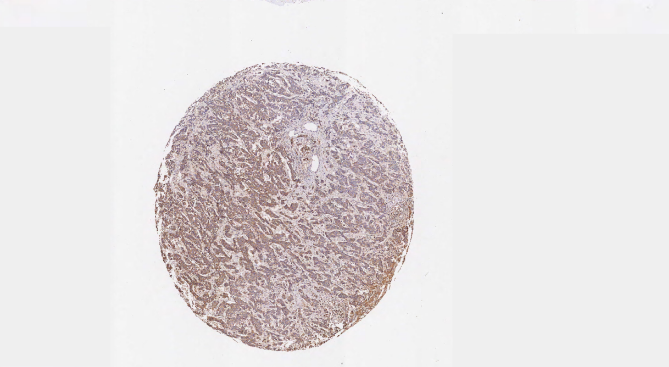


Figure 9B


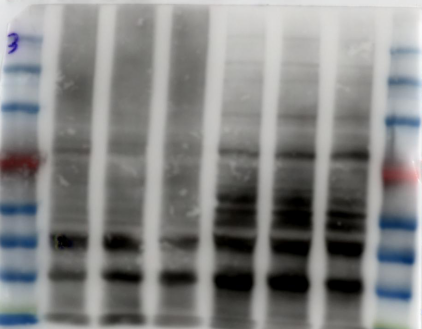

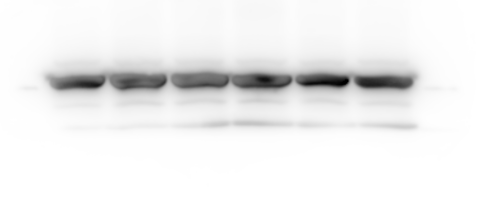


Figure 9C


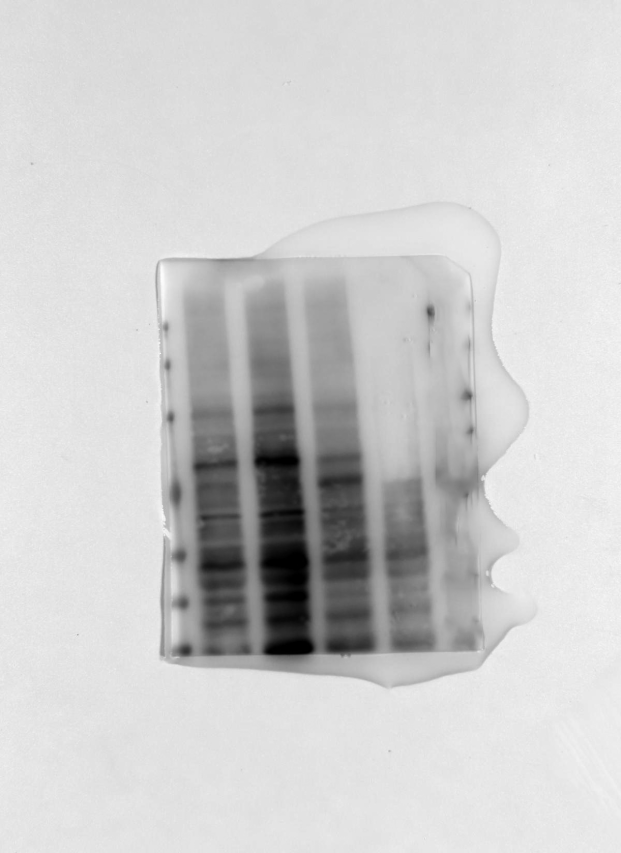

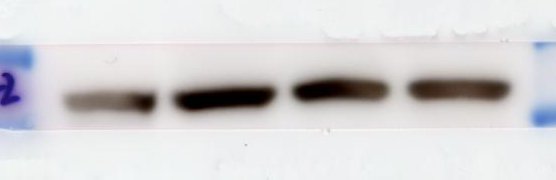


Figure 9D


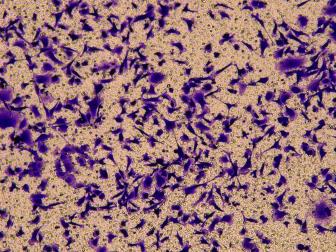

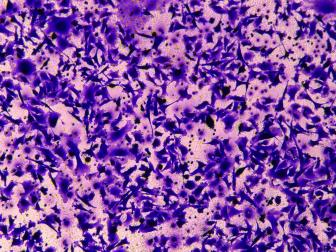


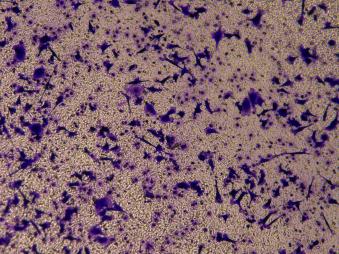

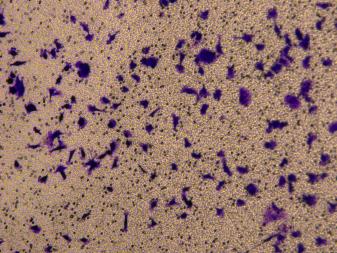

Supplement: Supplementary file 2 [file Table2.docx]
